# Transcriptome analysis of potato shoots, roots and stolons under nitrogen stress

**DOI:** 10.1038/s41598-020-58167-4

**Published:** 2020-01-24

**Authors:** Jagesh Kumar Tiwari, Tanuja Buckseth, Rasna Zinta, Aastha Saraswati, Rajesh Kumar Singh, Shashi Rawat, Vijay Kumar Dua, Swarup Kumar Chakrabarti

**Affiliations:** Indian Council of Agricultural Research-Central Potato Research Institute, Shimla, Himachal Pradesh 171001 India

**Keywords:** Transcriptomics, Abiotic

## Abstract

Potato crop requires high dose of nitrogen (N) to produce high tuber yield. Excessive application of N causes environmental pollution and increases cost of production. Hence, knowledge about genes and regulatory elements is essential to strengthen research on N metabolism in this crop. In this study, we analysed transcriptomes (RNA-seq) in potato tissues (shoot, root and stolon) collected from plants grown in aeroponic culture under controlled conditions with varied N supplies i.e. low N (0.2 milli molar N) and high N (4 milli molar N). High quality data ranging between 3.25 to 4.93 Gb per sample were generated using Illumina NextSeq500 that resulted in 83.60–86.50% mapping of the reads to the reference potato genome. Differentially expressed genes (DEGs) were observed in the tissues based on statistically significance (*p* ≤ 0.05) and up-regulation with ≥ 2 log_2_ fold change (FC) and down-regulation with ≤ −2 log_2_ FC values. In shoots, of total 19730 DEGs, 761 up-regulated and 280 down-regulated significant DEGs were identified. Of total 20736 DEGs in roots, 572 (up-regulated) and 292 (down-regulated) were significant DEGs. In stolons, of total 21494 DEG, 688 and 230 DEGs were significantly up-regulated and down-regulated, respectively. Venn diagram analysis showed tissue specific and common genes. The DEGs were functionally assigned with the GO terms, in which molecular function domain was predominant in all the tissues. Further, DEGs were classified into 24 KEGG pathways, in which 5385, 5572 and 5594 DEGs were annotated in shoots, roots and stolons, respectively. The RT-qPCR analysis validated gene expression of RNA-seq data for selected genes. We identified a few potential DEGs responsive to N deficiency in potato such as glutaredoxin, Myb-like DNA-binding protein, WRKY transcription factor 16 and FLOWERING LOCUS T in shoots; high-affinity nitrate transporter, protein phosphatase-2c, glutaredoxin family protein, malate synthase, CLE7, 2-oxoglutarate-dependent dioxygenase and transcription factor in roots; and glucose-6-phosphate/phosphate translocator 2, BTB/POZ domain-containing protein, F-box family protein and aquaporin TIP1;3 in stolons, and many genes of unknown function. Our study highlights that these potential genes play very crucial roles in N stress tolerance, which could be useful in augmenting research on N metabolism in potato.

## Introduction

Nitrogen (N) is an essential nutrient for plant growth and it is an integral component of several plant compounds. N fertilizers are used excessively to increase crop yield^1^. In potato, application of high dose of nitrogen is very common in fields to achieve high tuber yield. For example, in India, on an average 180–250 kg N/ha is applied in potato fields to get 30–40 tonnes per hectare tuber yield^2^. Of total N application, nearly half amount of N is utilized by potato crop^2^, whereas excessive N increases environmental pollution, deteriorates soil health and water quality, and also leads to higher cost of production^3^. Hence, understanding about genes involved in N metabolism in potato, especially low N stress, is essential to modulate plants to optimize the applied N. Several agronomic and soil-based approaches have been demonstrated in crops on N fertilization in plants including potato^4–6^. Unlike the model plant, *Arabidopsis thaliana*, and model crops rice and maize where rich source of genomics-based information is available on N metabolism research^3,7^, knowledge about potential genes involved in N deficiency and sufficiency in potato is very limited. Hence, identifying genes underlying N deficiency is essential to strengthen future research on N metabolism in potato.

Advancement has been made in N metabolism research in plants^1^. The N metabolic pathways are controlled by various genes, which express differently under varied N regimes. The key enzymes/genes involved in N metabolism (uptake, translocation, assimilation/utilization and remobilization) pathways are nitrate transporter (NRT), ammonium transporter, nitrate reductase, nitrite reductase, glutamine synthetase, glutamate synthase, glutamate dehydrogenase, asparagine synthetase, aspartate aminotransferase, alanine aminotransferase; and many other genes/regulatory molecules alter N pathways and determine gene expression as well as phenotypes^3^. Previous studies have investigated roles of nitrate transporters, signal transduction molecules and transcription factors in N metabolism in plants^3^. Moreover, RNA-sequencing technology has been proven to discover numerous genes/factors involved in N gene networks in several crops for multiple traits such as role of N starvation in rice^8,9^; identifying nitrogen deficiency genes in cucumber^10^; effect of N limitation condition in maize^7^, N use efficiency genes in *Brassica juncea*^11^, N responsive regulatory elements in potato^12^, N utilization genes in tea^13^ and so on.

Recently, we have proposed an integrated approach of breeding, genomics and physiology to improve NUE in potato by translating knowledge from other plants like *Arabidopsis thaliana*, rice, maize and wheat^14^. Furthermore, we have also demonstrated precision phenotyping of potato in aeroponic culture with accurate macro- and micro-nutrients supplies including minimum N dose while maintaining tuber yield^15^. With the availability of potato genome sequences^16^ and increasing sequencing facilities at low cost, it is now possible to unveil genes underlying N metabolism in potato. To our knowledge, in potato Gálvez *et al*.^12^ have identified N responsive genes and regulatory motifs in field-grown plants using RNA sequencing technology.

Hence, the aim of this study was to discover genes/regulatory elements associated with N deficiency (low N) versus sufficient N (high N, control) in potato plants grown in aeroponic culture under controlled conditions by RNA-sequence-based transcriptomes analysis. Differentially expressed genes (DEGs), heat map, Venn diagram, scatter plot, volcano plots, GO characterization, KEGG pathways analysis, motifs identification and potential genes to be involved in N metabolism pathways in potato were analyzed. Further, selected DEGs were validated by real-time qPCR analysis. Our results usher an insight on enrichment of genes in potato under N deficiency, which could be utilized for N metabolism related research in potato.

## Results

### Transcriptome data generation and processing

**Figure 1:**
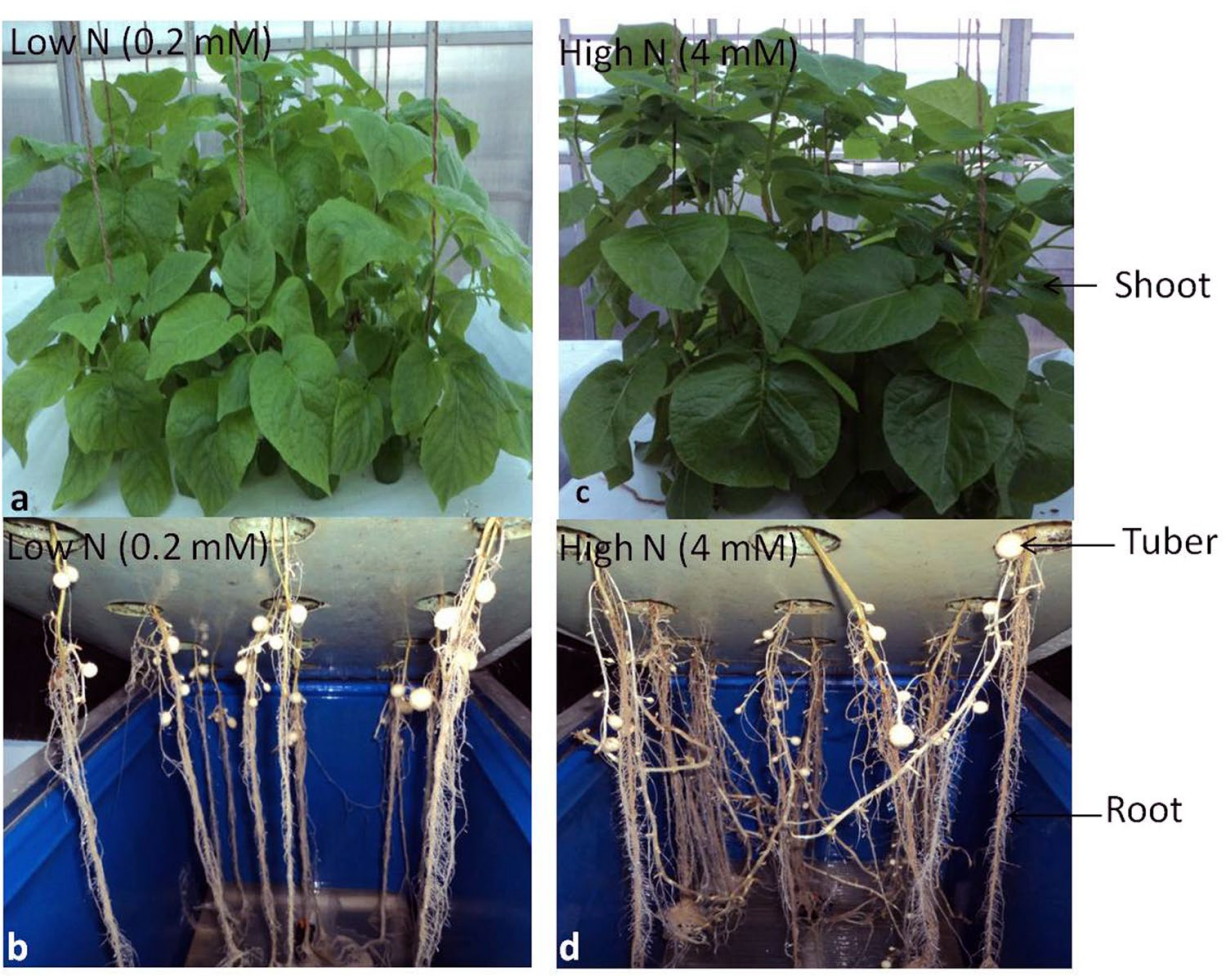
Phenotypes of potato plants grown in aeroponic culture with low N (0.2 milli Molar) and high N (4 milli Molar) supply showing shoots (**a,c**) and roots (**b,d**) biomass under different N regimes. (**a,b**: low N; **c,d**: high N).

A popular Indian potato variety Kufri Jyoti was grown in three replicates in aeroponic culture under controlled conditions with varied N supplies (low N: 0.2 milli molar N; and high N: 4 milli molar N) (Fig. 1). To obtain an overview of genes and regulatory elements involved in N metabolism in potato, especially low N stress versus high N (control), plant tissues (shoots, roots and stolons) were analyzed by RNA-sequencing. High quality data (QV > 20) ranging between 3.25 to 4.93 Gb per sample were generated using Illumina technology. The quality reads of all six tissues showed 83.60-86.50% mapping to the reference potato genome (Table S1 and Fig. S1).

### Differentially expressed genes (DEGs) analysis

**Figure 2:**
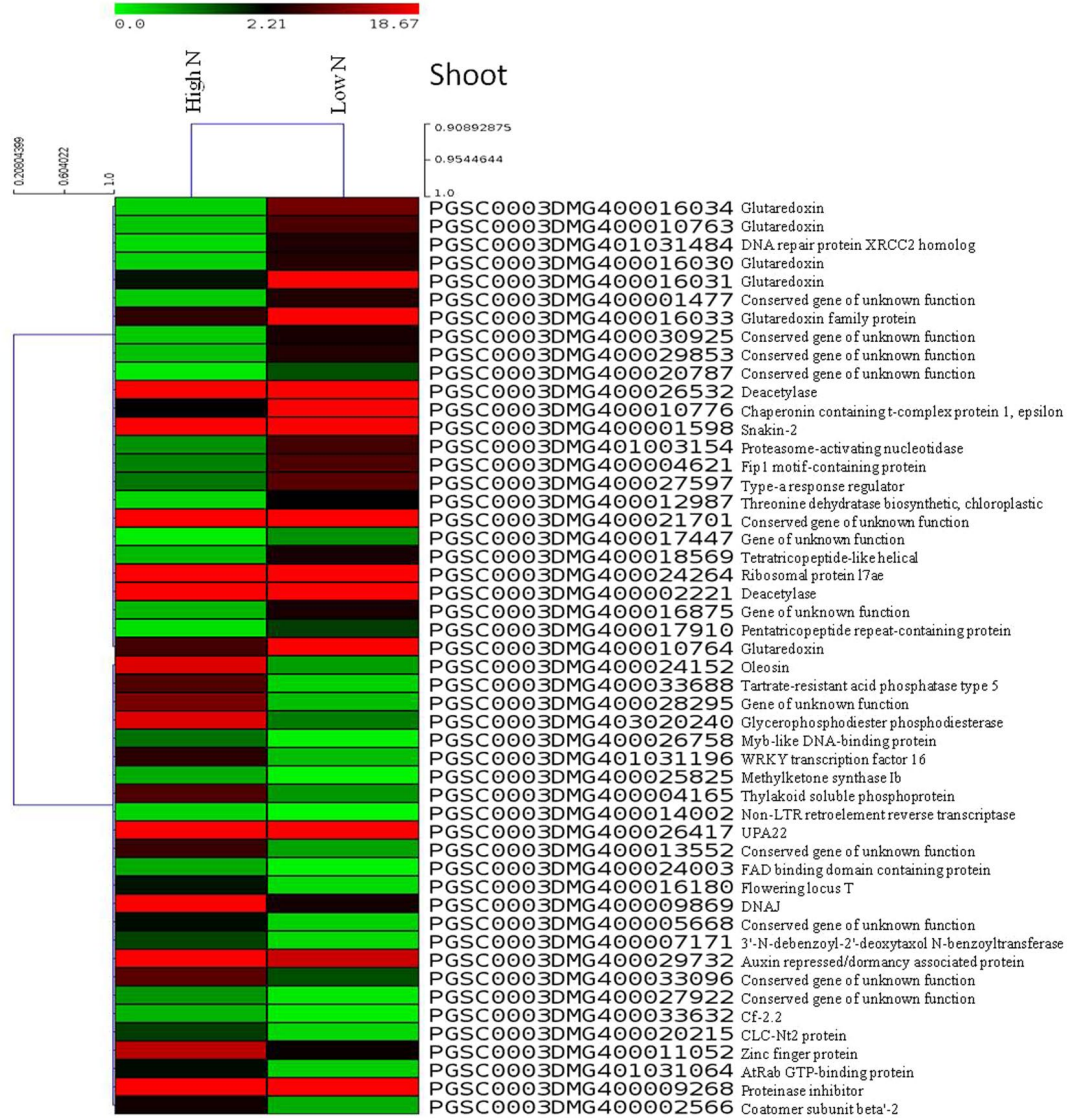
Heat maps of top 50 differentially expressed genes in shoots of potato plants grown in aeroponic culture with low N (0.2 mM) and high N (4 mM, control) supply. In the heat maps, each horizontal line refers to a gene. Relatively up-regulated genes are shown in red colour, whereas down-regulated genes are shown in green colour under low N stress compared to control (high N).

**Figure 3:**
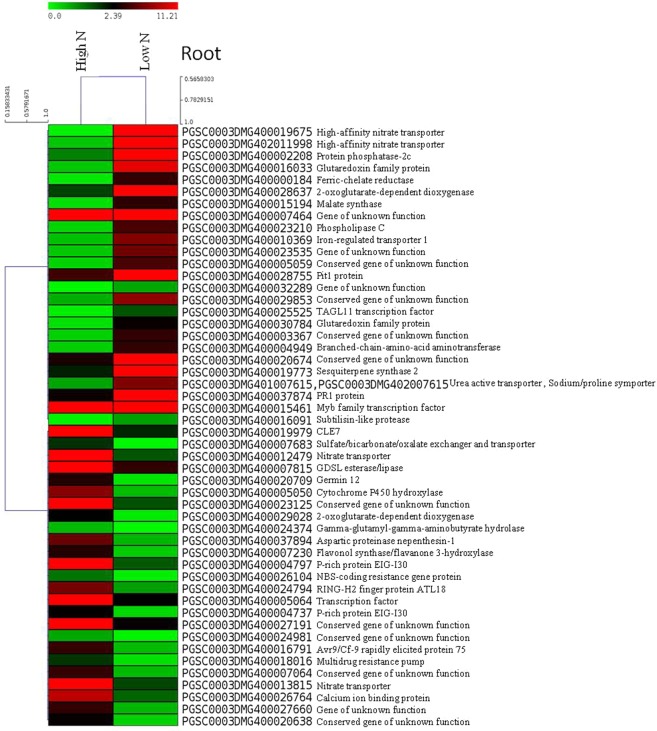
Heat maps of top 50 differentially expressed genes in roots of potato plants grown in aeroponic culture with low N (0.2 mM) and high N (4 mM, control) supply. In the heat maps, each horizontal line refers to a gene. Relatively up-regulated genes are shown in red colour, whereas down-regulated genes are shown in green colour under low N stress compared to control (high N).

**Figure 4:**
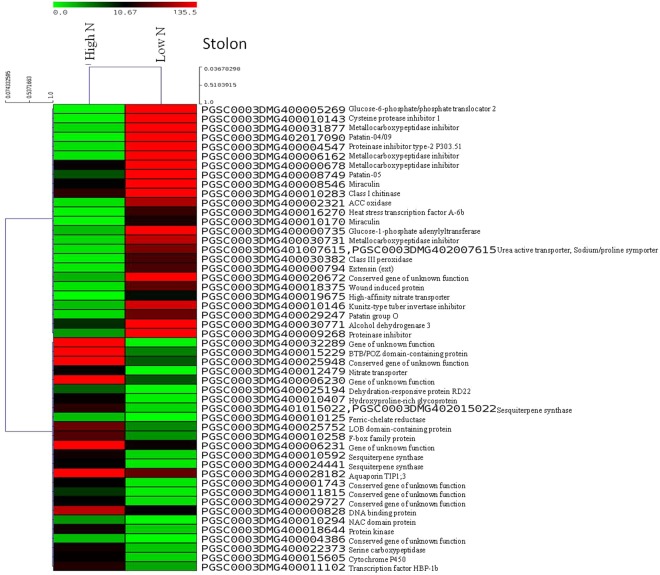
Heat maps of top 50 differentially expressed genes in stolons of potato plants grown in aeroponic culture with low N (0.2 mM) and high N (4 mM, control) supply. In the heat maps, each horizontal line refers to a gene. Relatively up-regulated genes are shown in red colour, whereas down-regulated genes are shown in green colour under low N stress compared to control (high N).

Individual transcriptomes were assembled and then DEGs were identified using cufflinks and cuffdiff, respectively. DEGs were analyzed separately for shoot, root and stolon tissues between low N stress and high N (control) (Table S2). Complete list of DEGs is provided in supplementary datasets (Table S3: shoots; Table S4: roots; and Table S5: stolons). Significant DEGs were identified based on the statistical significance (*p ≤ *0.05) and ≥ 2 log_2_ fold change (FC) for up-regulated, and ≤ −2 log_2_ FC values for down-regulated genes. In shoots, a total of 19730 genes were differentially expressed, of which significant DEGs were 761 (up-regulated) and 280 (down-regulated); whereas, of 20736 DEGs in roots, significant DEGs were 572 (up-regulated) and 292 (down-regulated). In stolons, of 21494 DEG, 688 and 230 DEGs were significantly up-regulated and down-regulated, respectively. In addition, many DEGs were exclusively expressed in the tissues. Heat maps of the top 50 DEGs are shown in Fig. 2 (shoots), Fig. 3 (roots), and Fig. 4 (stolons). Where, red and green colours represent up-regulated and down-regulated DEGs, respectively in low N compared to high N. Scatter plot (Fig. S2) and volcano plot (Fig. S3) show significant up-regulated and down-regulated DEGs in shoot, root and stolon tissues.

**Figure 5:**
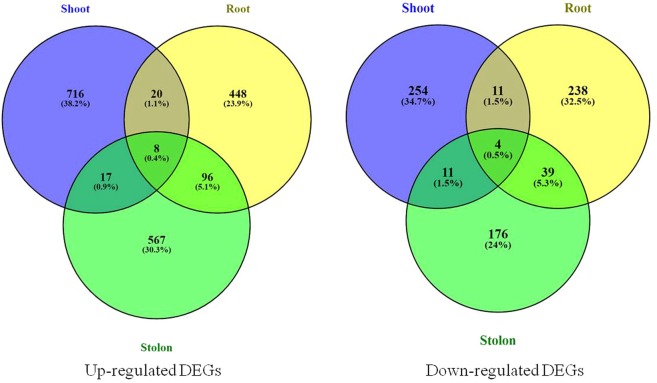
Venn diagrams of up-regulated and down-regulated DEGs showing distribution of tissues-specific and tissues-independent genes in potato plants (shoot, root and stolon).

Venn diagram analysis showed that only eight up-regulated and four down-regulated DEGs were common in shoot, root and stolon tissues (Fig. 5). In general, gene categories such as glutaredoxins, transcription factors (Myb, WRKY, LOB domain and heat shock transcription factors), nitrate transporters, aquaporin and ABC transporter, sulfate/bicarbonate/oxalate exchanger and transporter sat-1, urea active transporter, sodium/proline symporter, ferric-chelate reductase, miraculin, and methylketone synthase Ib were differentially expressed (up-/down-regulation) in all three tissues. Particularly, genes with known function namely extension (PGSC0003DMG400000783), ferric-chelate reductase (PGSC0003DMG400000184), GTP binding protein (PGSC0003DMG402012350), UPF0497 membrane protein (PGSC0003DMG400027047) were up-regulated, methylketone synthase Ib (PGSC0003DMG400025825), CLC-Nt2 protein (PGSC0003DMG400020215), stem-specific protein TSJT1 (PGSC0003DMG400012822) genes were down regulated in all three tissues. Besides, many genes showed tissue-specific response under N stress in potato (supplementary dataset: Table S15).

### Identification of potential genes under N stress

Selected top 20 significantly up-regulated ( ≥ 2.0 log_2_ FC; *p* < 0.05) and down-regulated ( ≤ −2.0 log_2_ FC; *p* < 0.05) DEGs under N stress are summarized in Table 1 (shoots), Table 2 (roots) and Table 3 (stolons). These DEGs recorded high levels of gene expression in all the tissues such as shoots (up-regulated between 4.61 to 2.72 log_2_ FC, and down-regulated between −4.31 to −2.35 log_2_ FC); roots (up-regulated: 6.96 to 3.12 log_2_ FC, and down-regulated: −5.67 to −2.83 log_2_ FC); stolons (up-regulated: 10.07 to 5.70 log_2_ FC, and down-regulated: −8.93 to −3.22 log_2_ FC).

**Table 1:** Selected top 20 differentially expressed genes (DEG) (*p* < 0.05) in potato shoots under low N stress versus high N (control).

SN	Gene name	Locus	Log_2_ fold change^#^	P value	Gene description	Gene Ontology (GO)^!^		
*Up-regulated*						Accession	Term name	Domain
1.	PGSC0003DMG400016034	chr04:7732721-7733339	4.6196	0.032	Glutaredoxin	GO:0009055	Electron transfer activity	MF
2.	PGSC0003DMG400010763	chr04:7654066-7654709	3.86229	0.010	Glutaredoxin	GO:0009055	Electron transfer activity	MF
3.	PGSC0003DMG401031484^*^	chr01:4374602-4379187	3.85194	0.031	DNA repair protein XRCC2 homolog	GO:0006281	DNA repair	BP
4.	PGSC0003DMG400016030	chr04:7745283-7746056	3.56649	0.012	Glutaredoxin	GO:0009055	Electron transfer activity	MF
5.	PGSC0003DMG400016031	chr04:7743340-7744028	3.48105	0.000	Glutaredoxin	GO:0009055	Electron transfer activity	MF
6.	PGSC0003DMG400001477	chr02:45141073-45141685	3.27025	0.037	Conserved gene of unknown function	GO:0016020	Membrane	CC
7.	PGSC0003DMG400016033	chr04:7736223-7736932	3.22378	0.000	Glutaredoxin family protein	GO:0009055	Electron transfer activity	MF
8.	PGSC0003DMG400030925	chr07:4558381-4561474	3.12325	0.003	Conserved gene of unknown function	GO:0005515	Protein binding	MF
9.	PGSC0003DMG400029853	chr10:58129139-58131600	3.06688	0.047	Conserved gene of unknown function	GO:0003677	DNA binding	MF
10.	PGSC0003DMG400020787	chr08:32325201-32327253	3.00156	0.004	Conserved gene of unknown function	NA	NA	NA
11.	PGSC0003DMG400026532	chr11:39737589-39747899	2.93537	0.000	Deacetylase	GO:0016020	Membrane	CC
12.	PGSC0003DMG400010776	chr05:11213911-11214833	2.88528	0.000	Chaperonin containing t-complex protein 1, epsilon subunit	GO:0005524	ATP binding	MF
13.	PGSC0003DMG400001598	chr01:87145670-87146801	2.82116	0.000	Snakin-2	GO:0006952	Defense response	BP
14.	PGSC0003DMG401003154	chr03:47065337-47065851	2.81106	0.016	Proteasome-activating nucleotidase	GO:0005524	ATP binding	MF
15.	PGSC0003DMG400004621^*^	chr12:60014011-60022309	2.77399	0.000	Fip1 motif-containing protein	NA	NA	NA
16.	PGSC0003DMG400027597^*^	chr06:34954418-34956541	2.75881	0.003	Type-a response regulator	GO:0005622	Intracellular	CC
17.	PGSC0003DMG400012987	chr09:4336456-4342614	2.74515	0.003	Threonine dehydratase biosynthetic, chloroplastic	GO:0006520	Cellular amino acid metabolic process	BP
18.	PGSC0003DMG400021701	chr03:33474459-33476134	2.73122	0.000	Conserved gene of unknown function	GO:0005759	Mitochondrial matrix	CC
19.	PGSC0003DMG400017447	chr05:12289815-12293142	2.726	0.014	Gene of unknown function	NA	NA	NA
20.	PGSC0003DMG400018569	chr11:42703065-42704595	2.7255	0.000	Tetratricopeptide-like helical	GO:0009451	RNA modification	BP
***Down-regulated***								
1.	PGSC0003DMG400024152	chr06:44102307-44102827	−4.31724	0.011	Oleosin	GO:0016020	Membrane	CC
2.	PGSC0003DMG400033688^*^	chr03:48558975-48561647	−4.27514	0.000	Tartrate-resistant acid phosphatase type 5	GO:0016311	Dephosphorylation	BP
3.	PGSC0003DMG400028295	chr10:55340167-55340737	−4.17885	0.026	Gene of unknown function	NA	NA	NA
4.	PGSC0003DMG403020240^*^	chr02:48163233-48167144	−3.87685	0.000	Glycerophosphodiester phosphodiesterase	GO:0008081	Phosphoric diester hydrolase activity	MF
5.	PGSC0003DMG400026758	chr04:3706321-3711165	−3.52714	0.004	Myb-like DNA-binding protein	GO:0003677	DNA binding	MF
6.	PGSC0003DMG401031196	chr04:54616204-54620206	−3.18115	0.008	WRKY transcription factor 16	GO:0005634	Nucleus	CC
7.	PGSC0003DMG400025825	chr01:85023387-85024892	−3.11856	0.045	Methylketone synthase Ib	NA	NA	NA
8.	PGSC0003DMG400004165	chr06:3451771-3452389	−3.02566	0.010	Thylakoid soluble phosphoprotein	NA	NA	NA
9.	PGSC0003DMG400014002	chr12:7372859-7383339	−3.0022	0.041	Non-LTR retroelement reverse transcriptase	GO:0090502	RNA phosphodiester bond hydrolysis, endonucleolytic	BP
10.	PGSC0003DMG400026417	chr09:59174647-59175464	−2.96379	0.000	UPA22	GO:0098807	Chloroplast thylakoid membrane protein complex	CC
11.	PGSC0003DMG400013552	chr07:40343169-40343952	−2.92426	0.004	Conserved gene of unknown function	GO:0016020	Membrane	CC
12.	PGSC0003DMG400024003^*^	chr04:23619207-23621616	−2.87291	0.048	FAD binding domain containing protein	GO:0055114	Oxidation-reduction process	BP
13.	PGSC0003DMG400016180^*^	chr11:3249173-3251562	−2.78112	0.033	Flowering locus T	NA	NA	NA
14.	PGSC0003DMG400009869	chr04:70887700-70888496	−2.55408	0.001	DNAJ	NA	NA	NA
15.	PGSC0003DMG400005668	chr03:58456136-58462029	−2.45442	0.002	Conserved gene of unknown function	GO:0006508	Proteolysis	BP
16.	PGSC0003DMG400007171	chr10:59411620-59412907	−2.41769	0.025	3’-N-debenzoyl-2’-deoxytaxol N-benzoyltransferase	GO:0016747	transferase activity, transferring acyl groups other than	MF
17.	PGSC0003DMG400029732	chr02:33667269-33668316	−2.41394	0.000	Auxin repressed/dormancy associated protein	NA	NA	NA
18.	PGSC0003DMG400033096	chr06:52073024-52073720	−2.40545	0.006	Conserved gene of unknown function	NA	NA	NA
19.	PGSC0003DMG400027922	chr04:19581182-19582907	−2.37338	0.026	Conserved gene of unknown function	NA	NA	NA
20.	PGSC0003DMG400033632	chr08:42364701-42367767	−2.35312	0.023	Cf-2.2	GO:0016020	Membrane	CC

^#^DEG analysis was performed between low N and high N (control); NA: Not Available. !Gene Ontology (GO) domains: BP = Biological Process, CC = Cellular Component, MF = Molecular Function.

*In the above selected genes, KEGG pathways (KO nos.) are available for seven genes: PGSC0003DMG401031484 (K10879 XRCC2/DNA-repair protein XRCC2); PGSC0003DMG400004621 (K14405 FIP1L1, FIP1/pre-mRNA 3’-end-processing factor FIP1); PGSC0003DMG400027597 (K14492 ARR-A/ two-component response regulator ARR-A family); PGSC0003DMG400033688 (K14379 ACP5/ tartrate-resistant acid phosphatase type 5 [EC:3.1.3.2]); PGSC0003DMG403020240 (K18696 GDE1/ glycerophosphodiester phosphodiesterase [EC:3.1.4.46]); PGSC0003DMG400024003 (K00103 GULO/ L-gulonolactone oxidase [EC:1.1.3.8]); and PGSC0003DMG400016180 (K16223 FT/ protein FLOWERING LOCUS T).

**Table 2:** Selected top 20 differentially expressed genes (DEG) (*p* < 0.05) in potato roots under low N stress versus high N (control).

SN	Gene name	Locus	Log_2_ fold change^#^	P value	Gene description	Gene Ontology^!^		
*Up-regulated*						Accession	Term name	Domain
1.	PGSC0003DMG400019675	chr11:41946329-41947803	6.96935	0.045	High-affinity nitrate transporter	GO:0016020	Membrane	CC
2.	PGSC0003DMG402011998	chr06:55526923-55528285	4.68973	0.000	High-affinity nitrate transporter	GO:0016020	Membrane	CC
3.	PGSC0003DMG400002208	chr08:36803306-36804175	4.50816	0.001	Protein phosphatase-2c	GO:0004722	Protein serine/ threonine phosphatase activity	MF
4.	PGSC0003DMG400016033	chr04:7736223-7736932	4.30678	0.007	Glutaredoxin family protein	GO:0009055	Electron transfer activity	MF
5.	PGSC0003DMG400000184	chr01:72491748-72495021	4.26991	0.001	Ferric-chelate reductase	GO:0055114	Oxidation-reduction process	BP
6.	PGSC0003DMG400028637	chr12:50107858-50110978	3.76324	0.000	2-oxoglutarate-dependent dioxygenase	GO:0055114	Oxidation-reduction process	BP
7.	PGSC0003DMG400015194^*^	chr03:50835984-50839603	3.58563	0.000	Malate synthase	GO:0016740	Transferase activity	MF
8.	PGSC0003DMG400007464	chr04:634862-635970	3.57342	0.000	Gene of unknown function	NA	NA	NA
9.	PGSC0003DMG400023210	chr10:46715043-46715807	3.54774	0.023	Phospholipase C	GO:0008081	Phosphoric diester hydrolase activity	MF
10.	PGSC0003DMG400010369	chr02:28280246-28281091	3.54431	0.006	Iron-regulated transporter 1	GO:0016020	Membrane	CC
11.	PGSC0003DMG400023535	chr05:50094386-50094932	3.54301	0.047	Gene of unknown function	NA	NA	NA
12.	PGSC0003DMG400005059	chr03:2249234-2252078	3.50499	0.027	Conserved gene of unknown function	GO:0016747	Transferase activity, transferring acyl groups other than	MF
13.	PGSC0003DMG400028755	chr07:6804296-6804982	3.44441	0.000	Pit1 protein	GO:0006952	Defense response	BP
14.	PGSC0003DMG400032289	chr00:37147945-37150150	3.29805	0.033	Gene of unknown function	NA	NA	NA
15.	PGSC0003DMG400029853	chr10:58129139-58131600	3.29542	0.024	Conserved gene of unknown function	GO:0003677	DNA binding	MF
16.	PGSC0003DMG400025525	chr11:16127865-16137240	3.25772	0.033	TAGL11 transcription factor	GO:0000977	RNA polymerase II regulatory region sequence-specific DNA	MF
17.	PGSC0003DMG400030784	chr06:40917982-40918948	3.22547	0.027	Glutaredoxin family protein	GO:0009055	Electron transfer activity	MF
18.	PGSC0003DMG400003367	chr09:20711524-20712601	3.20632	0.005	Conserved gene of unknown function	NA	NA	NA
19.	PGSC0003DMG400004949	chr04:66316676-66319860	3.12903	0.005	Branched-chain-amino-acid aminotransferase	GO:0009081	Branched-chain amino acid metabolic process	BP
20.	PGSC0003DMG400020674	chr12:5220523-5221009	3.11828	0.001	Conserved gene of unknown function	NA	NA	NA
***Down-regulated***								
1.	PGSC0003DMG400019979	chr07:49991771-49992124	−5.67445	0.02015	CLE7	NA	NA	NA
2.	PGSC0003DMG400007683	chr09:54234198-54239398	−4.434	0.01485	Sulfate/bicarbonate/oxalate exchanger and transporter sat-1	GO:1902358	Sulfate transmembrane transport	BP
3.	PGSC0003DMG400012479	chr08:479007-483551	−4.15594	0.00005	Nitrate transporter	GO:0005215	Transporter activity	MF
4.	PGSC0003DMG400007815	chr12:1866472-1869939	−4.03867	0.00005	GDSL esterase/lipase	GO:0016788	Hydrolase activity, acting on ester bonds	MF
5.	PGSC0003DMG400020709	chr01:79649685-79650749	−3.83927	0.0205	Germin 12	GO:0046872	Metal ion binding	MF
6.	PGSC0003DMG400005050	chr03:2449240-2450931	−3.48392	0.0047	Cytochrome P450 hydroxylase	GO:0055114	Oxidation-reduction process	BP
7.	PGSC0003DMG400023125	chr07:31908969-31909654	−3.4448	0.0004	Conserved gene of unknown function	GO:0005509	Calcium ion binding	MF
8.	PGSC0003DMG400029028^*^	chr11:10118207-10119335	−3.41982	0.03345	2-oxoglutarate-dependent dioxygenase	NA	NA	NA
9.	PGSC0003DMG400024374	chr03:4876744-4880852	−3.30241	0.0285	Gamma-glutamyl-gamma-aminobutyrate hydrolase	GO:0006541	Glutamine metabolic process	BP
10.	PGSC0003DMG400037894	chr04:64866740-64868048	−3.11435	0.00175	Aspartic proteinase nepenthesin-1	GO:0016787	Hydrolase activity	MF
11.	PGSC0003DMG400007230	chr02:30769141-30771030	−2.93025	0.0056	Flavonol synthase/ flavanone 3-hydroxylase	GO:0055114	Oxidation-reduction process	BP
12.	PGSC0003DMG400004797	chr08:52532858-52533558	−2.92293	0.0021	P-rich protein EIG-I30	NA	NA	NA
13.	PGSC0003DMG400026104	chr06:46731068-46738912	−2.90423	0.036	NBS-coding resistance gene protein	GO:0016301	Kinase activity	MF
14.	PGSC0003DMG400024794	chr01:77814015-77814779	−2.85057	0.00695	RING-H2 finger protein ATL18	GO:0016020	Membrane	CC
15.	PGSC0003DMG400005064	chr03:2341282-2344678	−2.83902	0.0001	Transcription factor	GO:0046983	Protein dimerization activity	MF
16.	PGSC0003DMG400004737	chr08:52807392-52808142	−2.76247	0.03925	P-rich protein EIG-I30	NA	NA	NA
17.	PGSC0003DMG400027191	chr05:48180807-48182363	−2.76161	0.0082	Conserved gene of unknown function	GO:0006979	Response to oxidative stress	BP
18.	PGSC0003DMG400024981	chr02:43251920-43256279	−2.74754	0.0141	Conserved gene of unknown function	NA	NA	NA
19.	PGSC0003DMG400016791	chr05:46313483-46314069	−2.74487	0.0392	Avr9/Cf-9 rapidly elicited protein 75	NA	NA	NA
20.	PGSC0003DMG400018016	chr07:4968687-4973344	−2.70617	0.02455	Multidrug resistance pump	GO:0016020	Membrane	CC

#DEG analysis was performed between low N and high N (control); NA: Not Available. !Gene Ontology (GO) domains: BP = Biological Process, CC = Cellular Component, MF = Molecular Function.

*In the above selected genes, KEGG pathways (KO nos.) are available for two genes: PGSC0003DMG400015194 (K01638 aceB, glcB/ malate synthase [EC:2.3.3.9]), and PGSC0003DMG400029028 (K06892 F6H1/ feruloyl-CoA ortho-hydroxylase [EC:1.14.11.-]).

**Table 3:** Selected top 20 differentially expressed genes (DEG) (*p* < 0.05) in potato stolons under low N stress versus high N (control).

SN	Gene name	Locus	Log_2_ fold change^#^	P value	Gene description	Gene Ontology^!^		
*Up-regulated*						Accession	Term name	Domain
1.	PGSC0003DMG400005269	chr05:42300216-42303680	10.0739	0.002	Glucose-6-phosphate/phosphate translocator 2	GO:0016020	Membrane	CC
2.	PGSC0003DMG400010143	chr03:49548298-49549151	9.36055	0.037	Cysteine protease inhibitor 1	GO:0010466	Negative regulation of peptidase activity	BP
3.	PGSC0003DMG400031877	chr00:32054672-32056195	9.11066	0.003	Metallocarboxypeptidase inhibitor	GO:0004180	Carboxypeptidase activity	MF
4.	PGSC0003DMG402017090	chr08:1555287-1556629	8.34974	0.043	Patatin-04/09	GO:0008152	Metabolic process	BP
5.	PGSC0003DMG400004547	chr03:50056093-50056945	8.32273	0.001	Proteinase inhibitor type-2 P303.51	GO:0010466	Negative regulation of peptidase activity	BP
6.	PGSC0003DMG400006162	chr07:44717565-44718414	8.09625	0.025	Metallocarboxypeptidase inhibitor	NA	NA	NA
7.	PGSC0003DMG400000678	chr07:44804949-44806430	8.09594	0.000	Metallocarboxypeptidase inhibitor	GO:0004180	Carboxypeptidase activity	MF
8.	PGSC0003DMG400008749	chr08:1435830-1439140	7.17945	0.000	Patatin-05	GO:0006952	Defense response	BP
9.	PGSC0003DMG400008546	chr12:55090840-55092326	7.11605	0.000	Miraculin	GO:0010951	Negative regulation of endopeptidase activity	BP
10.	PGSC0003DMG400010283	chr10:38265410-38265939	6.5655	0.000	Class I chitinase	GO:0008061	Chitin binding	MF
11.	PGSC0003DMG400002321^*^	chr07:32977277-32979126	6.07216	0.000	ACC oxidase	GO:0055114	Oxidation-reduction process	BP
12.	PGSC0003DMG400016270	chr06:40218131-40220250	6.01278	0.002	Heat stress transcription factor A-6b	GO:0003700	DNA-binding transcription factor activity	MF
13.	PGSC0003DMG400010170	chr03:49838682-49840071	6.00484	0.050	Miraculin	GO:0010951	Negative regulation of endopeptidase activity	BP
14.	PGSC0003DMG400000735	chr01:86092272-86097263	5.94054	0.000	Glucose-1-phosphate adenylyltransferase	GO:0005978	Glycogen biosynthetic process	BP
15.	PGSC0003DMG400030731	chr07:3198623-3200152	5.932	0.002	Metallocarboxypeptidase inhibitor	GO:0010951	Negative regulation of endopeptidase activity	BP
16.	PGSC0003DMG401007615	chr08:47514381-47518067	5.90116	0.000	Urea active transporter	NA	NA	NA
17.	PGSC0003DMG402007615	chr08:47514381-47518067	5.90116	0.000	Sodium/proline symporter	NA	NA	NA
18.	PGSC0003DMG400030382	chr06:57556038-57558160	5.89448	0.002	Class III peroxidase	GO:0042744	Hydrogen peroxide catabolic process	BP
19.	PGSC0003DMG400000794	chr04:59085132-59085620	5.7076	0.050	Extensin (ext)	NA	NA	NA
20.	PGSC0003DMG400020672	chr12:5232331-5232819	5.4688	0.002	Conserved gene of unknown function	NA	NA	NA
***Down-regulated***								
1.	PGSC0003DMG400032289	chr00:37147945-37150150	−8.9334	0.000	Gene of unknown function	NA	NA	NA
2.	PGSC0003DMG400015229	chr03:51465623-51468593	−7.03707	0.000	BTB/POZ domain-containing protein	GO:0006355	Regulation of transcription, DNA-templated	BP
3.	PGSC0003DMG400025948	chr01:85047797-85048333	−6.59358	0.000	Conserved gene of unknown function	GO:0016020	Membrane	CC
4.	PGSC0003DMG400012479	chr08:479007-483551	−6.16693	0.033	Nitrate transporter	GO:0005215	Transporter activity	MF
5.	PGSC0003DMG400006230	chr10:42813265-42813830	−5.03105	0.000	Gene of unknown function	NA	NA	NA
6.	PGSC0003DMG400025194	chr01:85774330-85776301	−4.76956	0.034	Dehydration-responsive protein RD22	NA	NA	NA
7.	PGSC0003DMG400010407	chr02:22351253-22353133	−4.43034	0.020	Hydroxyproline-rich glycoprotein	GO:0016020	Membrane	CC
8.	PGSC0003DMG401015022	chr07:46154267-46157856	−4.28363	0.010	Sesquiterpene synthase	NA	NA	NA
9.	PGSC0003DMG402015022	chr07:46154267-46157856	−4.28363	0.010	Sesquiterpene synthase	NA	NA	NA
10.	PGSC0003DMG400010125	chr03:43879401-43884284	−3.79246	0.023	Ferric-chelate reductase	GO:0055114	Oxidation-reduction process	BP
11.	PGSC0003DMG400025752	chr01:83704498-83706155	−3.74499	0.000	LOB domain-containing protein	NA	NA	NA
12.	PGSC0003DMG400010258	chr10:38340058-38342634	−3.54484	0.000	F-box family protein	GO:0005515	Protein binding	MF
13.	PGSC0003DMG400006231	chr10:42807585-42808186	−3.54054	0.000	Gene of unknown function	NA	NA	NA
14.	PGSC0003DMG400010592	chr07:46366478-46370711	−3.51052	0.005	Sesquiterpene synthase	GO:0008152	Metabolic process	BP
15.	PGSC0003DMG400024441	chr07:46310184-46311374	−3.42869	0.008	Sesquiterpene synthase	GO:0000287	Magnesium ion binding	MF
16.	PGSC0003DMG400028182	chr10:55272335-55274167	−3.38114	0.000	Aquaporin TIP1;3	GO:0016020	membrane	CC
17.	PGSC0003DMG400001743	chr09:6336960-6339479	−3.34861	0.015	Conserved gene of unknown function	NA	NA	NA
18.	PGSC0003DMG400011815	chr06:40610372-40612477	−3.29462	0.008	Conserved gene of unknown function	GO:0005215	Transporter activity	MF
19.	PGSC0003DMG400029727	chr02:33923113-33929372	−3.23751	0.018	Conserved gene of unknown function	NA	NA	NA
20.	PGSC0003DMG400000828	chr05:2094103-2097092	−3.22163	0.000	DNA binding protein	GO:0003677	DNA binding	MF

^#^DEG analysis was performed between low N and high N (control); NA: Not Available. !Gene Ontology (GO) domains (BP = Biological Process, CC = Cellular Component, MF = Molecular Function).

*In the above selected genes, KEGG pathways (KO nos.) are available for one gene only: PGSC0003DMG400002321 (K05933 E1.14.17.4/ aminocyclopropanecarboxylate oxidase [EC:1.14.17.4]).

Analysis of the DEGs across the tissues showed that glutaredoxin/glutaredoxin family genes were over expressed ( > 3 log_2_ FC) in both shoots and roots, and played an important role in N stress tolerance in potato. High-affinity nitrate transporter genes were also highly expressed in roots under N stress. Similarly, in stolon tissues ion transporters (e.g. urea active transporter, sodium/proline symporter) were highly up-regulated (>5 log_2_ FC), nitrate transporter and ion homeostasis genes like ferric-chelate reductase were down-regulated (< −3 log_2_ FC), and metallocarboxypeptidase inhibitor was over expressed (>5 log_2_ FC). In roots, ferric chelate reductase was up-regulated; however, sulfate/bicarbonate/ oxalate exchanger and transporter sat-1, nitrate transporter were under expressed (< −3 log_2_ FC). Other important genes like phospho-lipid related genes (e.g. tartrate-resistant acid phosphatase type 5, glycerophosphodiester phosphodiesterase) were under expressed in shoots, whereas GDSL esterase/lipase was down regulated (< −3 log_2_ FC) in roots; and protein phosphatase 2 C) were up-regulated in roots under N stress.

Transcription factors (TFs) play key roles in plant adaptation to stress metabolism. TFs (e.g. TAGL11) was up-regulated (>3 log_2_ FC) in roots; whereas, WRKY transcription factor 16 was under expressed (< −3 log_2_ FC) in shoots under N stress. TF like heat stress transcription factor A-6b was up-regulated (>5 log_2_ FC) in stolons. Besides, DNA binding proteins (e.g. BTB/POZ domain-, LOB domain-containing protein, F-box) were down-regulated (< −3 log_2_ FC) in stolons. Amino acid is an essential component of protein and its role was observed more in roots. The amino acid metabolism genes (e.g. malate synthase) were up-regulated (>3 log_2_ FC) in roots; whereas, gamma-glutamyl-gamma-aminobutyrate hydrolase was down-regulated (< −3 log_2_ FC) in roots. However, gene 2-oxoglutarate-dependent dioxygenase showed both up-regulation and down-regulation in roots under N stress.

Starch is the major food source of potato. A few classes of genes were only observed in stolon tissues. Starch/sugar metabolism related genes like glucose-6-phosphate/phosphate translocator 2 were highly up-regulated (>5 log_2_ FC) in stolons. Inhibitor proteins like cysteine protease inhibitor 1, tuber storage protein patatin-04/09, ethylene biosynthesis related genes ACC oxidase were highly over expressed (>5 log_2_ FC) in stolons. Terpenoids biosynthesis genes such as sesquiterpene synthase were under expressed (< −3 log_2_ FC) in stolons. Stress responsive genes like dehydration-responsive protein RD22, hydroxyproline-rich glycoprotein were under expressed (< −3 log_2_ FC) in stolons.

### Gene ontology (GO) characterization

All DEGs were functionally assigned with the GO terms, in which the molecular function GO domain was found to be highest in roots, shoots and stolons. Complete list and details are provided in supplementary datasets (shoots: Table S6; roots: Table S7 and stolons: Table S8) and result is summarized in Table S9. In shoots, 25142 molecular function followed by 18805 biological process and 17921 cellular component GO terms were observed. Whereas, in roots 26132 molecular function followed by 19402 biological process and 18210 cellular component GO terms were found. In stolon also, molecular function (27286) was predominant followed by biological process (19753) and cellular component (18578). Overall a few GO terms such as cell, cell part, membrane, membrane part, catalytic activity, binding, metabolic process, cellular process were highly enriched in both up-regulated and down-regulated DEGs under low N and high N in the tissues. WEGO plot showing the GO terms of DEGs in different tissues are depicted in supplementary figures (Fig. S4: shoots, Fig. S5: roots, and Fig. S6: stolons). A few GO terms were observed exclusively in certain tissues. Analysis across the tissues showed that GO terms such as molecular carrier activity (molecular function) and rhythmic process (biological process) were observed only in down-regulated shoots, whereas GO term locomotion (biological process) was observed only in down-regulated roots. None of the gene term was found exclusively only in up-regulated genes. This indicates role of various genes in N stress metabolism in potato.

### KEGG pathways analysis

The DEGs were processed in KAAS and classified them into 24 KEGG functional pathways categories, which include annotation of 5385, 5572 and 5594 DEGs in shoots, roots and stolons, respectively (supplementary datasets-Table S10, Table S11, Table S12; and supplementary file: Table S13). Overall, maximum annotated genes were found to be associated with KEGG pathways like signal transduction; translation; carbohydrate metabolism; transport and catabolism; folding, sorting and degradation; amino acid metabolism; energy metabolism; lipid metabolism; and environmental adaptation etc. The KEGG pathways such as lipid metabolism, metabolism of other amino acids, glycan biosynthesis and metabolism, metabolism of terpenoids and polyketides, biosynthesis of other secondary metabolites were highest in stolons than other tissues. Interestingly, carbohydrate metabolism, amino acid metabolism, signal transduction, membrane transport, and environmental adaptation KEGG pathways were highest in roots; whereas, energy metabolism and metabolism of cofactors and vitamins pathways were highest in shoots. KEGG pathways of N metabolism related genes are shown in Fig. S10. This signifies importance of various gene networks of N metabolism in potato.

### Conserved motifs analysis

Conserved motifs were analyzed in highly up-regulated (≥3 log_2_ FC) and down-regulated (≤−3 log_2_ FC) DEGs with known functions in the tissues using MEME (version 5.1.0) software. In shoots, six motifs were found with significant E values and all six were over-represented in glutaredoxin genes and some motifs in other genes (e.g. motif 1: TCCCATTTGGAWGTGTATCGAGCTCGTAAAYTATAGGGTTTGCT; motif 2: TGGTGAAAAT CACCACCGCRCTTGDTGCTCCCAACTTCATCACCATATCC; and motif 3: CTCATTAGCAC CWCCAACTAACTCTTTCCCTATAAATATTGCTGG). In roots, only motif 1 (AAGAGARRNA RWGRAAGAARAARVARA) and motif 2 (TTGGTGTTGSDVHTGGKGGA) were observed with significant E values and both motifs were over-represented in the genes studied. In stolon tissues, all six motifs were found with significant E value (e.g. motif 1: ATCYYTCYMWCWMY TCTCTWYTWTWTTCCWBATCTMCMTSWWMRCYTCTC; motif 2: TGRTKGCTYYWY SASMCATGCCKMMMCAAGAARCAKTRSWRARAWTKRAG; and motif 3: YTCYCCWT YNMCWMCYCHWYCTCCMCTYM) and motif 3 was over-represented in the genes studied, whereas metallocarboxypeptidase genes contained all six motifs. Motif details are shown in supplementary files (Fig. S7: shoot; Fig. S8: root; and Fig. S9: stolon).

### Validation by RT-qPCR analysis

Selected 12 genes (2 genes from each tissue of root, shoot, and stolon tissues of both low N and high N treatments) were validated by RT-qPCR analysis. Results were found in agreement with the RNA-seq-based gene expression pattern with minor variations in the log_2_ FC values (Table S14).

### Plant biomass, total chlorophyll and total N content analysis

**Figure 6:**
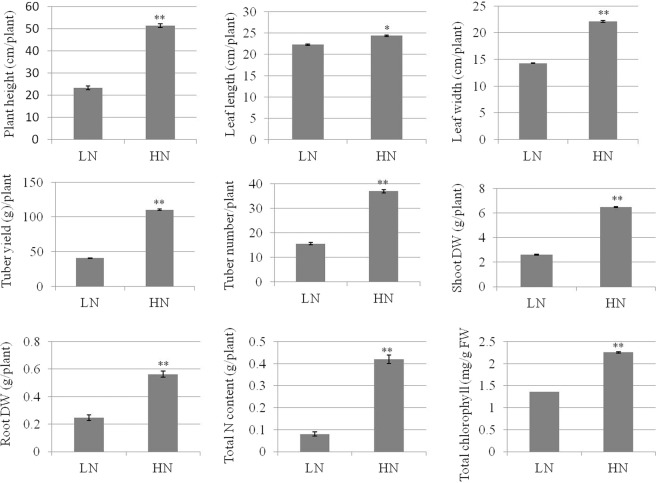
Plant biomass, tuber traits, total chlorophyll and total N content in potato plants of cv. Kufri Jyoti grown in aeroponic culture with contrasting N regimes till 90 days crop duration. DW: Dry weight, FW: Fresh weight, LN: Low N (0.2 mM N), HN: High N (4 mM N), Values on bar are mean ± standard error; * and ** indicate statistical significance at P ≤ 0.05 and P ≤ 0.01, respectively.

Phenotypic traits on per plant basis were estimated and showed higher biomass with high N supply. Traits such as plant height, leaf length, leaf width, tuber yield, tube number, root dry weight, shoot dry weight, total chlorophyll content and total N content were found significantly higher in high N than low N supplied plants (Fig. 6).

## Discussion

The present study provides an overview enrichment of genes associated with N metabolism in potato to provide adaptive strategies against N stress management through genomics intervention. Genes were analyzed in root, shoot and stolon tissues of potato plants grown in aeroponic culture for N metabolism such as uptake and utilization/assimilation and remobilization. This study revealed significant increase in plant biomass (plant height, leaf length, leaf width, shoot dry weight and root dry weight), fresh tuber yield, tuber number, total chlorophyll content and total N content with increasing N dose. Our results also concur with earlier findings on trends of variation in phenotypic traits under low N stress in potato^15^, for example, Gálvez *et al*.^12^ reported increase in plant biomass, tuber yield, leaf chlorophyll content on increasing N supply in potato.

RNA-seq approach has been applied in many crops for multiple traits to identify gene networks. In this study, we identified maximum number of up-regulated DEGs in shoots (761) followed by stolons (688) and roots (572), whereas maximum number of down-regulated DEGs were observed in roots (292), shoots (280) and stolons (230). In terms of fold change gene expression values, maximum up-regulation (10.073 log_2_ FC) and down-regulated (−8.933 log_2_ FC) were observed in stolon tissues followed by roots (6.969 and −5.674 log_2_ FC) and shoots (4.619 and −4.317 log_2_ FC). Besides, Venn diagram analysis unveiled tissue specific and common genes. The GO characterization shows predominance of molecular function in the tissues and some exclusive GO terms were also noticed. The GO terms like cell, cell part, membrane, membrane part, catalytic activity, binding, metabolic process, and cellular process were highly enriched in all the tissues. KEGG pathways analysis shows that the highest numbers of annotated genes belong to signal transduction, translation, and carbohydrate metabolism, and some genes in N metabolism. In motifs analysis, we identified more conserved motifs in shoots and stolons than roots, as reported earlier patatin gene by Gálvez *et al*.^12^ Further, RT-qPCR analysis of some selected genes validated the RNA-seq results. In potato, one study identified motifs associated with N genes in field-grown plants under with- and without-N supplies through RNA-seq approach^12^. Our study provides deeper insights into N stress responsive genes in potato, where plants were grown in aeroponic culture under controlled conditions with more precision on nutrients supply and plant growth as well as tuberization.

We identified a number of DEGs under low N stress in different potato tissues (shoot, root and stolon). Of which a few potential DEGs having higher gene expression (>2 log_2_ FC up-regulation or <−2 log_2_ FC down-regulation, as given in Tables 1, 2 and 3) are discussed here. Interestingly, glutaredoxins were the most highly up-regulated DEGs in shoots (also up-regulated in roots) during low N stress in potato compared to high N, and these were observed in all three tissues but more numbers of genes in shoots. A large number of glutaredoxin family proteins exist in plant, which are a group of oxidoreductases that control reactive oxygen species. Glutaredoxins families perform diverse functions in plants such as development, defense response, abiotic stress response (salt, drought, heavy metal, low nitrogen and temperature), redox signalling, flower development, hormonal regulation, ion homeostasis and adaptation to environments^17^. Another study shows differential expression of glutaredoxins in response to biotic and abiotic stresses during suberization in potato^18^, and overexpression of the CC-type glutaredoxin OsGRX6 affects hormone signalling and nitrogen status in rice^19^. Our study suggests that changes in expression of glutaredoxins in shoots play key roles in adaptation to N deficiency in potato.

In this study, transcription factors (Myb, WRKY, TAGL11, F-box, RING-H2 finger protein ATL18, heat shock TFs, heat stress transcription factor A-6b, BTB/POZ domain- and LOB domain-containing proteins) played crucial roles in regulation of genes during N deficiency in potato. Among them Myb, WRKY, LOB domain and heat shock TFs were common in all three tissues, whereas TFs like BTB/POZ domain and RING-H2 finger protein ATL18 were found in roots and stolons, and F-box TF was observed in shoot and stolon, and TAGL11 (root) and heat stress transcription factor A-6b (stolon) were tissue specific. The Myb and WRKY TFs were down-regulated in shoots under N stress; whereas, TAGL11 TF was up-regulated in roots under N stress. TFs are one of the key regulators in plant metabolism and constitute an important part of gene networks and signalling molecules for biotic and abiotic stress responses^20^. A study in maize shows that transcription factors GLK5, MADS64 and bZIP108, asparagine synthetase, protein kinase and a protein phosphatise are involved in N limitation conditions^21^. Besides, WRKY is one of the largest TFs gene families, which regulates signalling pathways and plays critical roles in plants in response to biotic and abiotic stresses^22^. The F-box proteins are encoded by a large number of gene families and function in various cellular processes including cell cycle, transcription and signalling molecules^23^. Furthermore, involvement of zinc finger protein 3 has been proven in salt stress and other osmotic response in *Arabidopsis thaliana*^24^. The roles of heat shock transcription factors (Hsfs) have been shown in response to various stresses, particularly adaptation to abiotic stresses. Transcriptomes and genomes have been investigated to study Hsfs in potato^25^. Besides, BTB/POZ domain-containing protein and LOB domain-containing protein were also highly down-regulated under low N stress in potato stolon tissues. Our study highlights that variation in the gene expression pattern of TFs and other binding proteins indicate their implication in N stress mechanism in potato.

Another gene FLOWERING LOCUS T was down-regulated under N deficiency exclusively in shoots only and implies to early plant maturity under N stress condition in potato. The FLOWERING LOCUS T protein is the main component of signalling molecules for flowering in plants, as studied in *Arabidpsis thaliana*, rice and tomato. Navarro *et al*.^26^ demonstrate that potato flowering and tuber development are controlled by FT-like paralogues namely StSP3D and StSP6A, which respond to different environmental cues. A study shows that CONSTANS molecules regulate StSP6A gene-driven tuberization in potato^26^. Another plant peptide hormones like CLAVATA3(CLV3)/EMBRYO SURROUNDING REGION-related (CLE7) family was the most down-regulated under N stress in roots only. The CLE molecules have been showed to play vital role in signalling for meristematic differentiations of roots and shoots, and play diverse roles in developmental process particularly symbiosis, parasitism and response to abiotic stress^27^. The varied gene expression of these genes indicates that these genes are important in potato under N deficiency.

Among the N metabolism genes, high-affinity nitrate transporters were the most down-regulated in roots under low N and suggest their crucial roles in N uptake mechanism for adaptation to N stress in potato. Overall, nitrate transporters were present in all three tissues, showing importance of N uptake in potato. Given that nitrate transporters perform very essential roles in N uptake in plants and they include mainly four gene families NRT1 (NPF), NRT2, CLC, and SLAC1/SLAH^28^. Nitrate transporters function for nitrogen uptake and root system architecture, protein storage, source to sink relationship, ionic balance, response to biotic and abiotic stresses, and carbon-nitrogen balance. Several reviews discussed the roles of nitrate transporters in regulation of plant growth and development and a potential target for marker or genomics-assisted breeding, genetic manipulation via genome editing or transgenic technologies^29,30^.

Down-regulation of aquaporin TIP1;3 gene was observed in stolons that showed its role in N stress in potato. In general, aquaporin and ABC transporter genes were found in all three tissues. The aquaporins are commonly known as water channel proteins, which regulate movement of water in plants. Aquaporins play important role in movement of solutes, small molecules and metal ions in response to biotic and abiotic stresses in plants^31^. Besides the urea transporters of *Arabidopsis thaliana*, several other transporters are known in plants like aquaporins and ABC transporters^32^. Furthermore in this study, genes like sulfate/bicarbonate/oxalate exchanger and transporter sat-1, urea active transporter, sodium/proline symporter, ferric-chelate reductase were also up-regulated or down-regulated in all the tissues. The genes such as sulfate/bicarbonate/oxalate exchanger and transporter sat-1 were down-regulated in roots, whereas urea active transporter, sodium/proline symporter were up-regulated in stolons under N stress. Previous study also found changes in sulphate-related genes in potato under varied N supplies^12^. Besides, ferric-chelate reductase and iron-regulated transporter 1 were down-regulated under N stress in potato roots. Role of ferric reductase oxidase enzyme is to reduce ferric Fe (III) into ferrous Fe (II) and thus play important roles in Fe homeostasis in plants^33^. Nevertheless, plant root architecture plays an important role in nutrient uptake in crops especially in potato^34^, where the underground plant part tuber is economically important. Our results indicate potential roles of these N metabolism associated genes especially transporters under N stress tolerance in potato.

Starch is the main component of potato tubers and has driven attentions for food as well as non-food/commercial uses. Despite numerous researches in potato biotechnology, detailed information on gene network in starch metabolism is limited^35^. In this study, glucose-6-phosphate/phosphate translocator 2 (GPT2) was the most up-regulated gene under N stress in stolons and it substantiates roles of carbohydrate metabolism related genes in potato tuberization. Moreover, this gene was found (up-regulation) in both root and stolon tissues, which could be associated with tuber development. Signalling role of GPT2 has been observed in seeds, seedlings and mature leaves of *Arabidopsis thaliana* in response to environments^36^. Glucose-6-phosphate is translocated via GPT during starch biosynthesis, in which inorganic phosphate is released or used as substrate during pentose phosphate pathways. To illustrate, a plastidic GPT has been purified from maize endosperm and corresponding cDNA was isolated from pea roots and potato tubers^37^. Another gene like glucose-1-phosphate adenylyltransferase was highly up-regulated exclusively in stolon tissues only under N stress, which is involved in starch and sucrose metabolism in potato^35^. Moreover, genes like malate synthase, branched-chain-amino-acid aminotransferase and 2-oxoglutarate-dependent dioxygenase were observed in only in root and stolon tissues but had varied fold change. In particular, malate synthase and branched-chain-amino-acid aminotransferase genes were highly up-regulated in roots, while 2-oxoglutarate-dependent dioxygenase gene was both up-regulated and down-regulated in roots under N stress. Role of 2-oxoglutarate-dependent dioxygenase gene *SlF3HL* has been confirmed recently for chilling stress tolerance in tomato^38^. Malate synthase plays an important role in starch synthesis in potato^39^, while branched-chain amino acid aminotransferase enzyme catalyzes conversion of branched-chain amino acids and α-ketoglutarate into branched chain α-keto acids and glutamate^40^. Thus, our study implicates that starch metabolism associated genes play vital role in potato tuberization process.

In addition, cytochrome P450 hydroxylase, gamma-glutamyl-gamma-aminobutyrate hydrolase, and flavonol synthase/flavanone 3-hydroxylase were either up- or down-regulated only in root and stolon tissues, but they were highly down-regulated in roots under N stress. The cytochrome P450 hydroxylases function in a variety of metabolic pathways in plants and involved in the jasmonic acid and ethylene signaling pathways, enhances plant resistance to biotic and abiotic stresses in soybean^41^ and under N stress in cucumber^10^. The gamma-glutamyl-gamma-aminobutyrate hydrolase catalyzes amino acid metabolism (gamma aminobutyric acid, GABA). The study shows that GABA concentration increases under various biotic and abiotic stress conditions in plants such as temperature, salinity, dehydration, low oxygen, mechanical damage etc; and play key roles in plant development during C:N balance^42^. Another study shows role of flavanone 3-hydroxylase in biosynthesis of phenolics (taxifolin and catechin) in spruce to confer defense against bark beetle and fungus associates^43^. Role of flavonol-specific genes like flavonol synthase/flavanone 3-hydroxylase has also been investigated in phenylpropanoid biosynthesis pathways in cucumber, which shows higher anthocyanin content under N deficiency^10^. The ACC oxidase was over-expressed in stolons under N stress and its role in ethylene biosysnthesis is well known under N limitation in cucumber^10^. The roles of these genes strengthen their importance in potato under N stress.

Some more genes like miraculin was found in all tissues, whereas patatin-05 and class III peroxidise were observed in root and stolon, and class I chitinase gene was differentially expressed in stolon only. All these genes were highly up-regulated in stolons under N stress. Patatin is the major protein of potato tubers. Reduced activities of defence-related enzymes like chitinase, chitosanase and peroxidase were reported in *Arabidopsis thaliana* at low N^44^. In stolons, dehydration-responsive protein RD22, hydroxyproline-rich glycoprotein and sesquiterpene synthase were highly down-regulated under N stress in potato. Recently, dehydration-responsive protein RD22 has been demonstrated for role under salt stress in soybean seedlings^45^. Hydroxyproline-rich glycoproteins function in plant cell wall in response to pathogens attack, as its role has been investigated in resistance to downy mildew in pearl millet^46^. Sesquiterpene synthase has been reported in many plants to play key roles in terpeniod metabolites synthesis and provide adaptation to adverse conditions under biotic and abiotic stresses in *Santalum album*^47^. Thus, these genes play vital roles in adaptation to N deficiency in potato.

Several other genes like protein kinases and phospholipids showed differential expression under N deficiency in potato. These genes were either over- or under-expressed in the tissues specific response such as tartrate-resistant acid phosphatases (shoot); phospholipase C (root); glycerophosphodiester phosphodiesterases, thylakoid soluble phosphoprotein, and histone deacetylase (shoot and root); cysteine protease inhibitor 1, metallocarboxypeptidase inhibitor, and proteinase inhibitor type-2 (root and stolon); and methylketone synthase Ib (root, shoot and stolon). The tartrate-resistant acid phosphatases (TRAcPs) and glycerophosphodiester phosphodiesterases were highly down-regulated under N stress in shoots. The TRAcPs, known as purple acid phosphatases (PAPs), have been identified in plants, animals and fungi, and particularly in *Arabidopsis thaliana* under phosphate starvation^48^. Glycerophosphodiester phosphodiesterase plays an important role in lipid metabolism, which releases inorganic phosphorous from phospholipids during P starvation^49,50^. One GDSL esterase/lipase gene was down-regulated genes under N stress in potato roots. A *Gossypium hirsutum* GDSL esterase/lipase functions as hydrolytic enzyme and involved in ovule and fibre development and plays a key role in seed development in *Arabidopsis thaliana*^51^. The phospholipase C gene was up-regulated under N stress in potato roots, and a previous study has characterized isoforms of phosphoinositide-specific phospholipase C in potato^52^. The methylketone synthase Ib and thylakoid soluble phosphoprotein genes were down-regulated in shoots and the protein phosphatase-2c was highly up-regulated in roots under N stress. The plant protein serine/threonine phosphatases (PP1/PP2A and PP2C) are the major families found in plants and animals, which function in kinase-associated protein phosphatase signalling pathways. The role of ABI1/ABI2, PP2C enzymes, has been envisaged in signal transduction of abscisic acid pathway in *Arabidopsis thaliana*; and alfalfa PP2C family functions as a negative regulator in plant mitogen-activated protein kinase pathways^53^. The inhibitors like cysteine protease inhibitor 1, metallocarboxypeptidase inhibitor and proteinase inhibitor type-2 P303.51 were up-regulated under N stress in stolons. Role of cysteine proteinase inhibitor has been envisaged in plants for growth and developmental, defense and stress response^54^. A complex network of proteases and protease inhibitors has been observed during multiple functions related to time and space in various biological pathways in plant life cycle^55^. Metallocarboxypeptidase inhibitors have been found in cDNA libraries of the wounded tissues of leaves treated with abscisic acid^56^. Histone deacetylase play key roles in regulation of gene expression of histone deacetylation in response to biotic and abiotic stresses in plants^57^. Thus, our study unveils many gene networks playing very crucial roles in potato during N stress tolerance metabolism.

## Conclusions

A large number of potential genes were discovered in this study in potato in response to N deficiency. Some of these genes showed very high up-regulation or down-regulation under N stress such as glutaredoxins, TFs (Myb, WRKY, BTB/POZ domain, LOB domain and F-box), high-affinity nitrate transporter/nitrate transporter, sodium/proline symporter, glucose-6-phosphate/phosphate translocator 2, cysteine protease inhibitor 1, metallocarboxypeptidase inhibitor, dehydraton responsive protein RD22, hydroxyproline-rich glycoprotein, ACC oxidase, and sesquiterpene synthase so on. In future, more investigations would be required in field-grown plants to observe transcripts changes in response to contrasting N regimes. Furthermore, functional validation of these potential genes via transgenic manipulation would also be essential. Taken together, the present study has greatly improved our knowledge on enrichment of gene networks and regulatory elements involved in N metabolism pathways in potato, which would necessarily strengthen research on N metabolism in potato in future.

## Methods

### Plant materials and nitrogen treatments

A potato variety Kufri Jyoti (KJ), N-inefficient^2,15^, was used in this study. *In vitro* plants were maintained at Indian Council of Agricultural Research-Central Potato Research Institute, Shimla (31.1048°N, 77.1734°E 2,276 m above mean sea level), Himachal Pradesh, India. Plants were grown in three replicates in aeroponic culture with two N treatments (low N: 0.2 mM N; and high N: 4 mM N, control) and other micro- and micro-nutrients remain same as described by Tiwari *et al*.^15^. N was supplied as NO_3_^−^ and NH_4_^+^ forms in both treatments. Plant tissues (shoots, roots and stolons) from both low N and high N were collected in three replicates after 30 days (roots, and shoots i.e. leaves) and 40 days (stolons, when stolon initiation/ tuberization were started) of planting in aeroponic culture. The collected six tissues (KJ-HighN Shoot, KJ-LowN Shoot, KJ-HighN Root, KJ-LowN Root, KJ-HighN Stolon, and KJ-LowN Stolon) were snap-frozen in liquid nitrogen and stored at −80 °C for transcriptomes sequencing. The tissue from three biological replicates was pooled for single sample for transcriptomes sequencing.

### Total RNA isolation and Illumina NextSeq500 PE library preparation

Total RNA was isolated from plant samples (shoots, roots and stolons of both low N and high N) using modified c-TAB and Lithium Chloride method^58^. The qualities and quantities of the isolated RNA samples were checked on 1% denaturing RNA agarose gel and NanoDrop (ThermoFisher Scientific, Wilmington, Delaware USA), respectively. RNA-seq paired end sequencing libraries were prepared from the QC passed RNA samples using Illumina TruSeq Stranded mRNA sample prep kit following the manufacturer instructions (Illumina, San Diego, CA, USA). Briefly, mRNA was enriched from the total RNA using poly-T attached magnetic beads, followed by enzymatic fragmentation, 1^st^ strand cDNA conversion using SuperScript II and Act-D mix to facilitate RNA dependent synthesis. The 1^st^ strand cDNA was then synthesized to second strand using second strand mix. The dscDNA was then purified using AMPure XP beads followed by A-tailing, adapter ligation and then enriched by limited no of PCR cycles. The PCR enriched libraries were analyzed on 4200 Tape Station system (Agilent Technologies, Santa Clara, CA, USA) using high sensitivity D1000 Screen tape as per the manufacturer instructions. The brief bioinformatics work flow is depicted in Fig. S1.

### Cluster generation and sequencing

After obtaining the Qubit 3.0 (ThermoFisher Scientific, Waltham, Massachusetts, USA) concentrations for the libraries and the mean peak sizes from Agilent Tape Station profiles, the PE illumina libraries were loaded onto NextSeq500 for cluster generation and sequencing. Paired-end sequencing allows the template fragments to be sequenced in both the forward and reverse directions on NextSeq500. The kit reagents were used in binding of samples to complementary adapter oligos on paired-end flow cell. The adapters were designed to allow selective cleavage of the forward strands after re-synthesis of the reverse strand during sequencing. The copied reverse strands were then used to sequence from the opposite end of the fragment.

### RNA sequencing and high quality read statistics

The raw data were processed to obtain high quality clean reads using Trimmomatic v0.38 to remove adapter sequences, ambiguous reads (reads with unknown nucleotides “N” larger than 5%), and low-quality sequences (reads with more than 10% quality threshold (QV) < 20 phred score). A minimum length of 50 nt (nucleotide) after trimming was applied. After removing the adapter and low quality sequences from the raw data, high quality reads were obtained. These high quality (QV > 20), paired-end reads were used for reference-based reads mapping. Parameters considered for filtration were as follows: *i*) SLIDINGWINDOW: Sliding window trimming of 10 bp, cutting once the average quality within the window falls below a threshold of 20, *ii*) LEADING: Cut bases off the start of a read, if below a threshold quality of 20, and *iii*) TRAILING: Cut bases off the end of a read, if below a threshold quality of 20.

### Reads mapping to the reference potato genome

The reference genome of *Solanum tuberosum* Group Phureja DM1-3, with a genome size of 840 Mb and the associated annotations were downloaded from the Spud DB database of the potato genome sequence^16^. The download links for genome was http://solanaceae.plantbiology.msu.edu/data/potato_dm_v404_all_pm_un.fasta.zip and for annotation was http://solanaceae.plantbiology.msu.edu/data/PGSC_DM_V403_genes.gff.zip. The high quality reads were mapped to the reference genome using TopHat v2.1.1 with default parameters^59^.

### Differential gene expression analysis

Cufflinks v2.2.1 program assembles transcriptomes from RNA-seq data and quantifies their expression. The individual gtf files of the transcriptomes were used for differential gene expression analysis using cuffdiff by blind dispersion method^60^. There are total of 39,028 protein coding genes present in annotation file of *Solanum tuberosum* Group Phureja DM1-3. Differential gene expression analysis was performed using cuffdiff version 2.2.1 between low N and high N (control) for shoots, roots and stolons. FPKM values were used to calculate the log fold change as log_2_ (FPKM_experimental/FPKM_control). Log_2_ FC values greater than zero were considered up-regulated whereas less than zero were down-regulated along with P-value threshold of 0.05 for statistically significant results. Venn diagrams of up-regulated and down-regulated DEGs were prepared using Venny 2.1 tool to analyze tissues-specific and tissues-independent genes for all three tissues^61^.

### Heat map

An average linkage hierarchical cluster analysis was performed with the top 50 DEGs using multiple experiments viewer (MeV v4.9.0)^62^. The heatmap shows level of gene abundance. Levels of expression are represented as log_2_ ratio of gene abundance between low N and high N (control) samples. Heatmaps were constructed using the log-transformed and normalized value of genes based on Pearson uncentered distance and average linkage method. In heatmap, each horizontal line refers to a gene. The color represents the logarithmic intensity of the expressed genes. Relatively higher gene expression values are shown in red colour and lower expression are shown in green colour.

### Scatter plot

The Eurofins proprietary R scripts were used to depict graphically the representing expression of genes in two distinct conditions of each sample combination i.e. low N and high N (control). It helps to identify DEGs in one sample with respect to another and also allows comparing two values associated with genes. In scatter plot, each dot represents a gene. The vertical position of each gene represents its expression level in the control (high N) samples while the horizontal position represents its expression level in low N samples. Thus, genes that fall above the diagonal are over-expressed and genes that fall below the diagonal are under-expressed as compared to their median expression level in experimental grouping of the experiment.

### Volcano plot

The Eurofins proprietary R scripts were used to depict the graphical representation and distribution of DEGs which were found in low N as well as high N samples. The volcano plot arranges DEGs along dimensions of biological as well as statistical significance. The red block on the right side of zero represents the up regulated genes whereas green block on the left side of zero represents significant down regulated genes. The Y-axis represents the negative log of p-value (*p* < 0.05) of the performed statistical test where data points with low p-values (highly significant) appearing towards the top of the plot. Grey block shows the non-DEGs.

### GO analysis

The GO annotations of the DEGs were obtained from the Ensembl Plants database for *Solanum tuberosum*. The GO annotations were categorized into up-regulated, down-regulated, expressed in both and exclusive DEGs. The information on number of genes was assigned into three main GO domains (biological process, cellular component, and molecular function). The bar plots, depicting the GO distribution, were obtained through the WEGO portal (http://wego.genomics.org.cn/cgi-bin/wego/index.pl)^63^.

### KEGG pathways analysis

The functional annotations of the DEGs were carried out against the curated KEGG GENES database using KAAS (KEGG Automatic Annotation Server-. (http://www.genome.jp/kegg/ko.html)^64^. The KEGG orthology (KO) database of Nightshade family was used as the reference for pathways mapping. The result contains KO assignments and automatically generated KEGG pathways using KAAS BBH (bidirectional best hit) method against available database.

### Conserved motifs analysis

Selected highly up-regulated ( ≥ 3 log_2_ fold change) and down-regulated ( ≤ −3 log_2_ fold change) DEGs with known function in shoots, roots and stolons were analysed using MEME (version 5.1.0) software^65^.

### Real time – quantitative polymerase chain reaction (RT-qPCR) analysis

Selected twelve differentially expressed genes (two genes from each shoots, roots and stolons from both low N and high N) were validated by RT-qPCR analysis. The coding sequences of the selected genes were downloaded from the potato genome sequence database (http://solanaceae.plantbiology.msu.edu/pgsc_download.shtml), and RT-qPCR primers were designed using IDT PrimerQuest Tool (https://eu.idtdna.com/Primerquest/Home/Index) following default parameters, as summarised in Table S6. The same samples were used RNA isolation using RNeasy Mini Kit (Qiagen, Venlo, Limburg, Netherlands) and cDNA synthesis using TaqMan Reverse Transcription Reagent (Applied Biosystems, New Jersey, USA). RT-PCR analysis was performed using Power SYBR Green PCR Master Mix in ABI PRISM HT7900 (Applied Biosystems Warrington, UK) following temperature/time profile 50 °C/2 min; 95 °C/10 min; and 40 cycles of 95 °C/15 s, 60 °C/1 min, and 72 °C/30 s with an internal standard potato ubiquitin-ribosomal protein gene (*ubi3*; L22576) as described in Tiwari *et al*.^66^.

### Plant biomass, total chlorophyll and N content analysis

Phenotypic traits, plant biomass, total chlorophyll and total N contents were measured as procedures described by Tiwari *et al*.^15^. Plants were grown for full crop cycle till 90 days crop duration to measure these traits. Observations were recorded on per pant basis in three replications for plant height (cm), leaf length (cm), leaf width (cm), tuber yield (g/plant), tuber number, total N content, total chlorophyll content (mg/g fresh weight), root dry weight (g) and shoot dry weight (g). Data were analysed by one way analysis of variance (ANOVA) using XLSTAT 2018.5 at least level of significance (*p* ≤ 0.05).

## Supplementary information

Suppl. Information file.

Dataset 1.

Dataset 4.

Dataset 5.

Dataset 6.

Dataset 2.

Dataset 3.

Dataset 7.

Dataset 8.

Dataset 9.

Dataset 10.

## Data Availability

The RNA-sequence data has been deposited with the NCBI database the Bioproject PRJNA529319; SRA Accession nos. SRR8846457, SRR8846458, SRR8846456, SRR8846453, SRR8846454 and SRR8846455; and BioSamples: SAMN11264445, SAMN11264444, SAMN11264442, SAMN11264441, SAMN11264440 and SAMN11264443.
