# Supplementary material for: Transcriptome analysis of potato shoots, roots and stolons under nitrogen stress
Source: Sci Rep. 2020 Jan 24;10:1152. doi: 10.1038/s41598-020-58167-4 (PMC6981199; doi:10.1038/s41598-020-58167-4)

Transcriptome analysis of potato shoots, roots and stolons under nitrogen stress

Jagesh Kumar Tiwari[1]*, Tanuja Buckseth[1], Rasna Zinta[1], Aastha Saraswati[1], Rajesh Kumar Singh[1], Shashi Rawat[1], Vijay Kumar Dua[1] and Swarup Kumar Chakrabarti[1]

Table S1.

a) **Quantification of isolated RNA sample using NanoDrop/Qubit Fluorometer**

Sr. No.	Sample ID	NanoDrop Readings (ng/µl)	NanoDrop OD $A_{260/280}$	NanoDrop OD $A_{260/230}$	Remark
1	KJ-HighN Shoot	2169.7	2.08	1.84	QC Pass
2	KJ-LowN Shoot	2727.3	2.07	1.74	QC Pass
3	KJ-HighN Root	2674.0	2.11	2.11	QC Pass
4	KJ-LowN Root	2301.6	2.15	2.18	QC Pass
1	KJ-HighN Stolon	414.2	2.12	2.12	QC Pass
2	KJ-LowN Stolon	256.6	2.17	2.02	QC Pass

b) **. High Quality Read statistics**

SL Num.	Sample Name (actual)	Sample Name (used during analysis)	No. of Filtered PE Reads	Total no. of bases	Data in Gb
1	KJ-HighN Shoot	KJHighNShoot	32,700,271	4,925,926,696	~4.93
2	KJ-LowN Shoot	KJLowNShoot	21,568,403	3,247,338,938	~3.25
3	KJ-HighN Root	KJHighNRoot	26,116,647	3,933,178,605	~3.93
4	KJ-LowN Root	KJLowNRoot	28,709,852	4,323,011,009	~4.32
5	KJ-HighN Stolon	KJHighNStolon	27,837,248	4,191,492,927	~4.19
6	KJ-LowN Stolon	KJLowNStolon	25,380,387	3,821,162,497	~3.82

c) **Mapping statistics**

Sl. Num.	Sample	Mapping %
1	KJHighNShoot	86.00
2	KJLowNShoot	85.90
3	KJHighNRoot	86.50
4	KJLowNRoot	84.10
5	KJHighNStolon	85.10
6	KJLowNStolon	83.60

[1] Indian Council of Agricultural Research-Central Potato Research Institute, Shimla, Himachal Pradesh – 171001, India.
*Corresponding author's email: jageshtiwari@gmail.com

Table S2. Number of differentially expressed genes (DEG) ($p<0.05$) under low N stress versus high N (control) in potato.

SN	Description	Tissues		
		Shoots	Roots	Stolons
1.	Up-regulated	761	572	688
2.	Down-regulated	280	292	230
3.	Exclusive in low N	114	115	364
4.	Exclusive in high N (control)	128	161	123
5.	Expressed in both	1041	864	918
6.	Expressed in both (total)	19,730	20,736	21,494

DEG were analyzed between low N versus High N (control) in all tissues (shoot, root and stolon)

Table S9. Gene Ontology statistics of DEG in potato cv. Kufri Jyoti grown in aeroponics with low N and high N (control) treatments.

Description		Genes		
		Biological Process	Cellular Component	Molecular Function
Shoots	Up-regulated	768	666	1,041
	Down-regulated	281	259	391
	Exclusive in low N	40	50	58
	Exclusive in high N	40	37	59
	Expressed in both	18,805	17,921	25,142
Roots	Up-regulated	654	417	848
	Down-regulated	326	195	434
	Exclusive in low N	43	50	61
	Exclusive in high N	58	54	65
	Expressed in both	19,402	18,210	26,132
Stolons	Up-regulated	772	607	1,087
	Down-regulated	290	205	347
	Exclusive in low N	175	152	241
	Exclusive in high N	47	51	52
	Expressed in both	19,753	18,578	27,286

Gene expression was analyzed between low N versus High N (control) in all the tissues (shoots, roots and stolons).

Table S13. KEGG pathway classification of DEG in the different tissues of potato cv. Kufri Jyoti grown in aeroponics with low N and high N (control) supply

KEGG pathways	Genes		
	Shoots	Roots	Stolons
i. Metabolism			
Carbohydrate metabolism	481	494	489
Energy metabolism	300	267	293
Lipid metabolism	301	298	307
Nucleotide metabolism	100	101	101
Amino acid metabolism	330	339	332
Metabolism of other amino acids	142	155	157
Glycan biosynthesis and metabolism	108	110	111
Metabolism of cofactors and vitamins	212	208	204
Metabolism of terpenoids and polyketides	148	166	173
Biosynthesis of other secondary metabolites	145	191	192
Xenobiotics biodegradation and metabolism	75	89	92
ii. Genetic Information Processing			
Transcription	213	220	216
Translation	527	532	536
Folding, sorting and degradation	421	437	436
Replication and repair	129	136	127
iii. Environmental Information Processing			
Membrane transport	25	31	30
Signal transduction	646	683	677
Signaling molecules and interaction	1	2	2
iv. Cellular Processes			
Transport and catabolism	385	386	388
Cell growth and death	276	285	275
Cellular community - eukaryotes	56	60	60
Cellular community - prokaryotes	54	50	50
Cell motility	40	40	40
v. Organismal Systems			
Environmental adaptation	270	292	306
Total	5385	5572	5594

Table S14. Validation of differentially expressed genes by RT-qPCR analysis in potato cv. Kufri Jyoti for low N stress tolerance

SN	Gene name	RT primers	Gene expression (Log_2 fold change)[#]		Gene description
			RT-qPCR	RNA-seq	
Shoots					
1.	PGSC0003DMG400016034	F: ACATCCAAATGGGAAGCAAATG<br>R: AGCACCTCCAACTAACTCTTTC	4.16	4.6196	Glutaredoxin
2.	PGSC0003DMG400010763	F: CAAATGGGAAGCAAATGGAGAA<br>R: CTCATTTGCACCACCAACTAAC	3.02	3.86229	Glutaredoxin
3.	PGSC0003DMG400024152	F: GATCGATTACGCGAGGGATAAG<br>R: ACCCTTAATCTGCTGCTGTG	-4.25	-4.31724	Oleosin
4.	PGSC0003DMG400033688	F: GTGGAACGTCTTCTTCCTATCC<br>R: CGGGCTTTCGGTATCACTTAT	-4.12	-4.27514	Tartrate-resistant acid phosphatase type 5
Roots					
5.	PGSC0003DMG400019675	F: GATGCACCATAACAGCATCAAA<br>R: GGTGTTACATTAGGCGGAGTAG	6.08	6.96935	High-affinity nitrate transporter
6.	PGSC0003DMG402011998	F: GCTGAGTACTTCTTCGACAGATT<br>R: CCTCCAAATGGACGAGCTAAA	4.28	4.68973	High-affinity nitrate transporter
7.	PGSC0003DMG400019979	F: AGGAGTATACGAGGCGATCTT<br>R: TAGTTGGAGGCAAAGAGTGATG	-5.12	-5.67445	CLE7
8.	PGSC0003DMG400007683	F: TGGCGTTTAGCTCACAGTATC<br>R: AACTCCTTCCAACTGCTATTCC	-4.11	-4.434	Sulfate/bicarbonate/oxalate exchanger and transporter sat-1
Stolons					
9.	PGSC0003DMG400005269	F: GATTCCCTTTGCTATTGCTGTG<br>R: GCCACCACCCACCATATAAA	9.63	10.0739	Glucose-6-phosphate/phosphate translocator 2
10.	PGSC0003DMG400031877	F: TGTTCTCTTGACGACCACTTTAG<br>R: GGTGCAATAATCGTTACAAGTTCC	8.70	9.11066	Metallocarboxypeptidase inhibitor
11.	PGSC0003DMG400015229	F: CAAAGAGAAGGTGCAACGAAAG<br>R: GGGCAGAGATAAAGAGGACAAA	-6.64	-7.03707	BTB/POZ domain-containing protein
12.	PGSC0003DMG400025194	F: CACACAGTTAGCAAGAGCAAAG<br>R: GACTCCATTCAGAGGTATCCAAG	-4.24	-4.76956	Dehydration-responsive protein RD22

[#]Gene expression analysis was performed between low N versus high N (control) treated samples.

Figure S1: Bioinformatics Workflow of RNA-seq. analysis

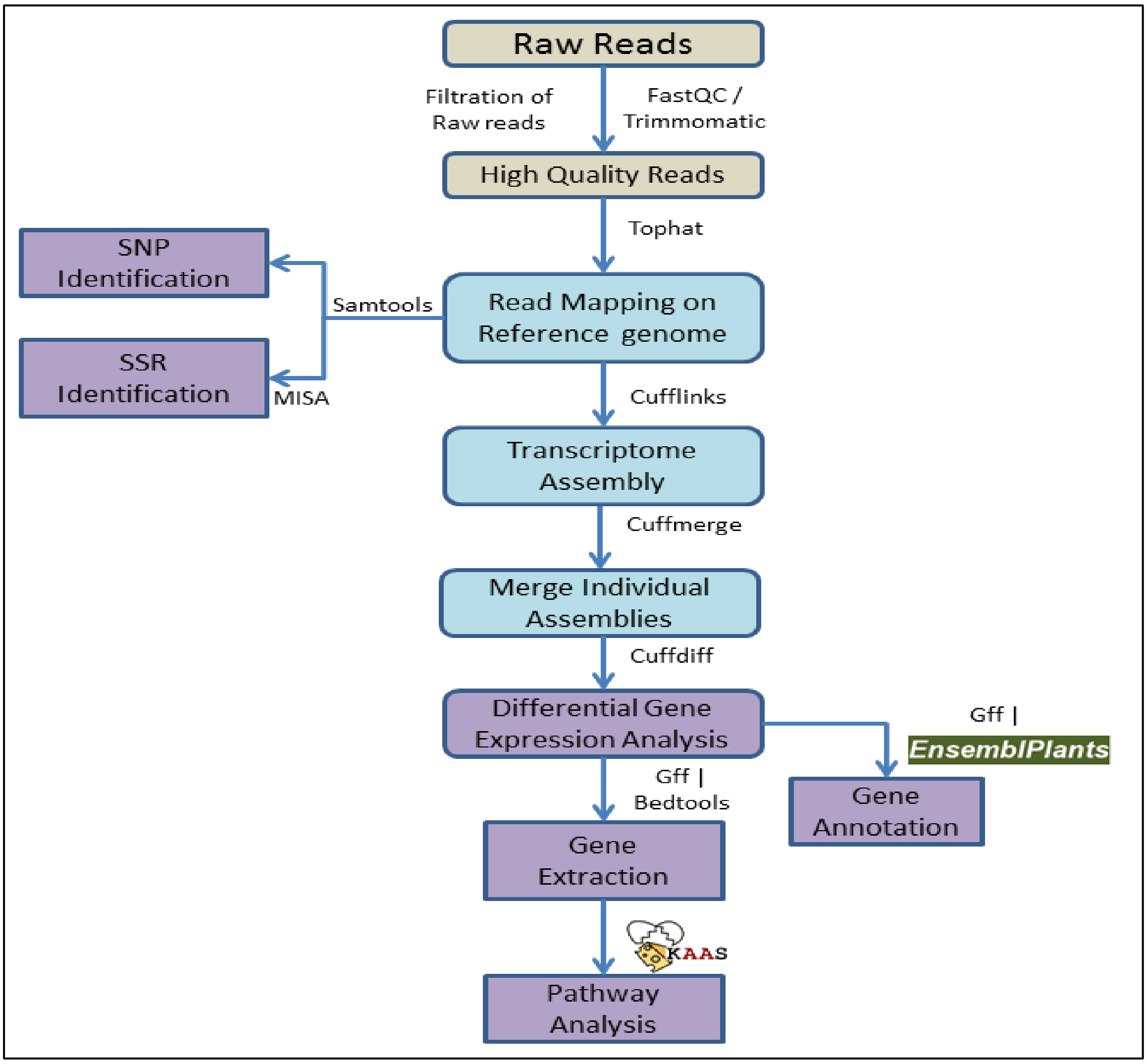

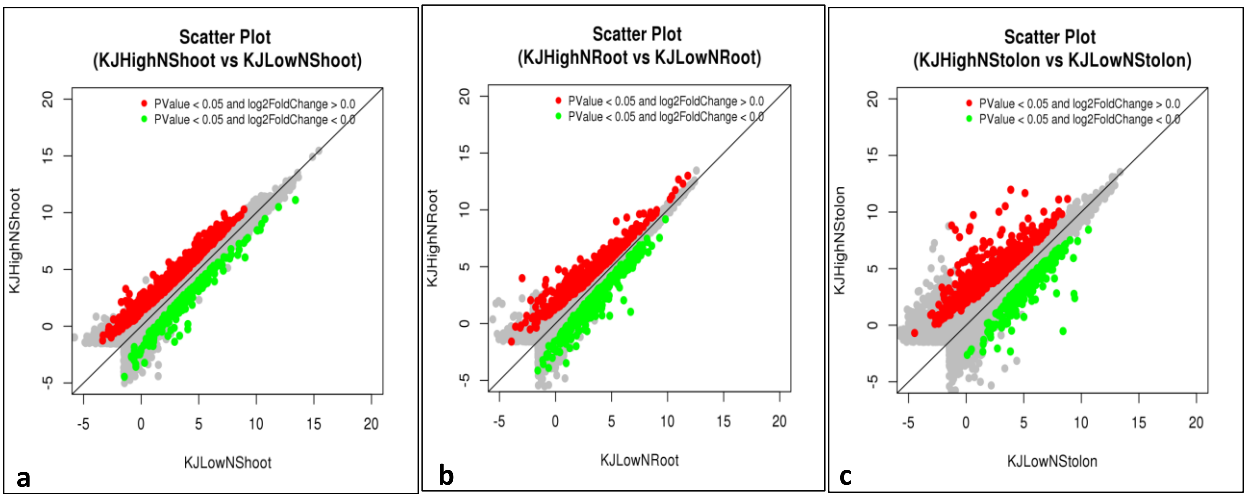

Figure S2: Scatter plot of the differentially expressed genes in potato cv. Kufri Jyoti plants grown in aeroponics with low N and high N (control) supply: a) shoots, b) roots, and c) stolons. Red and green dots represent the up-regulated and the down-regulated genes, respectively in low N compared to high N (control) samples.

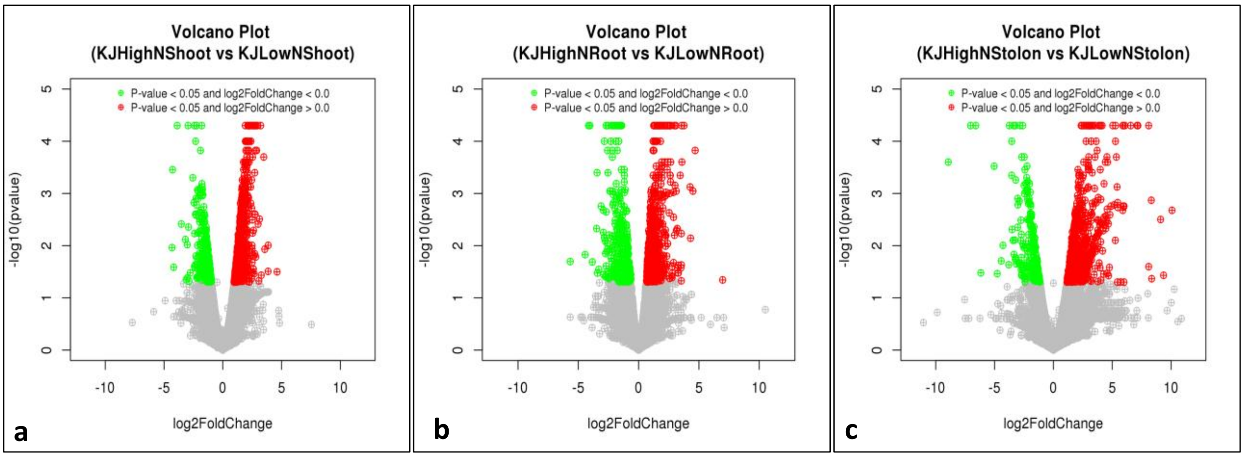

Figure S3: Volcano plot of differentially expressed genes in potato cv. Kufri Jyoti plants grown in aeroponics with low N and high N (control) supply: a) shoot, b) root, and c) stolon. Red and green dots represent the up-regulated and the down-regulated genes, respectively in low N compared to high N (control) samples.

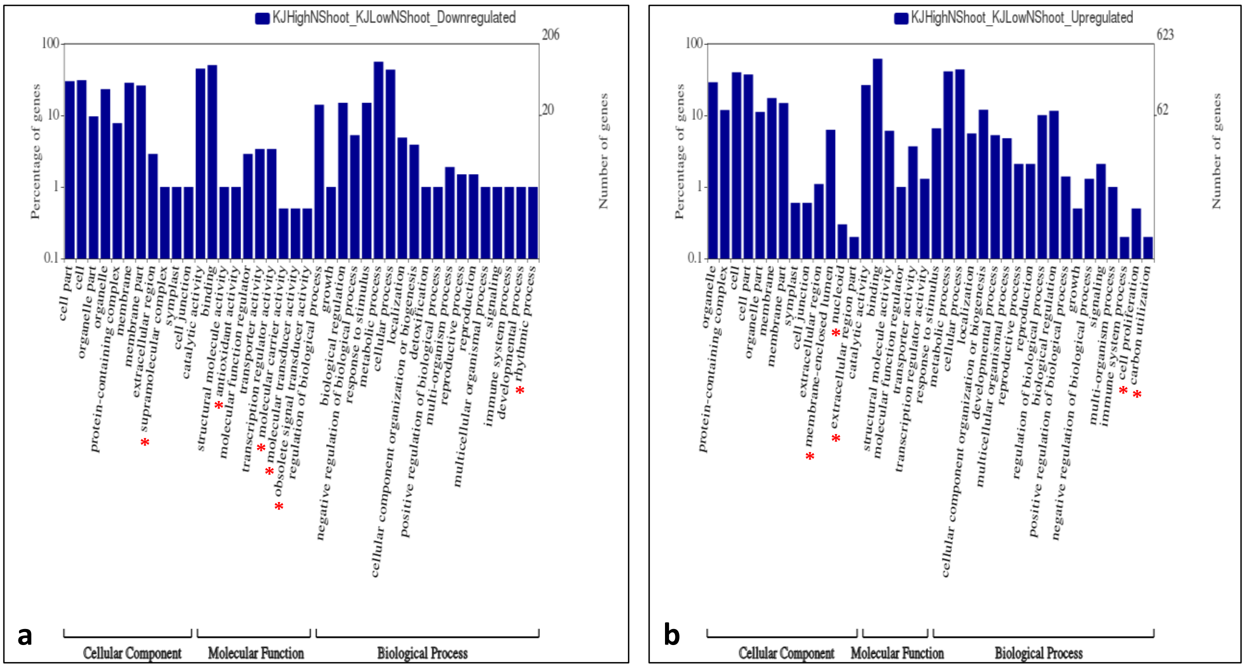

Figure S4. WEGO plot showing the gene ontology (GO) of differentially expressed genes in potato shoots grown in aeroponics with low N and high N (control) supply. a) down-regulated genes, and b) up-regulated genes. Red asterisk mark indicates exclusive GO term observed in either down-regulated or up-regulated DEGs.

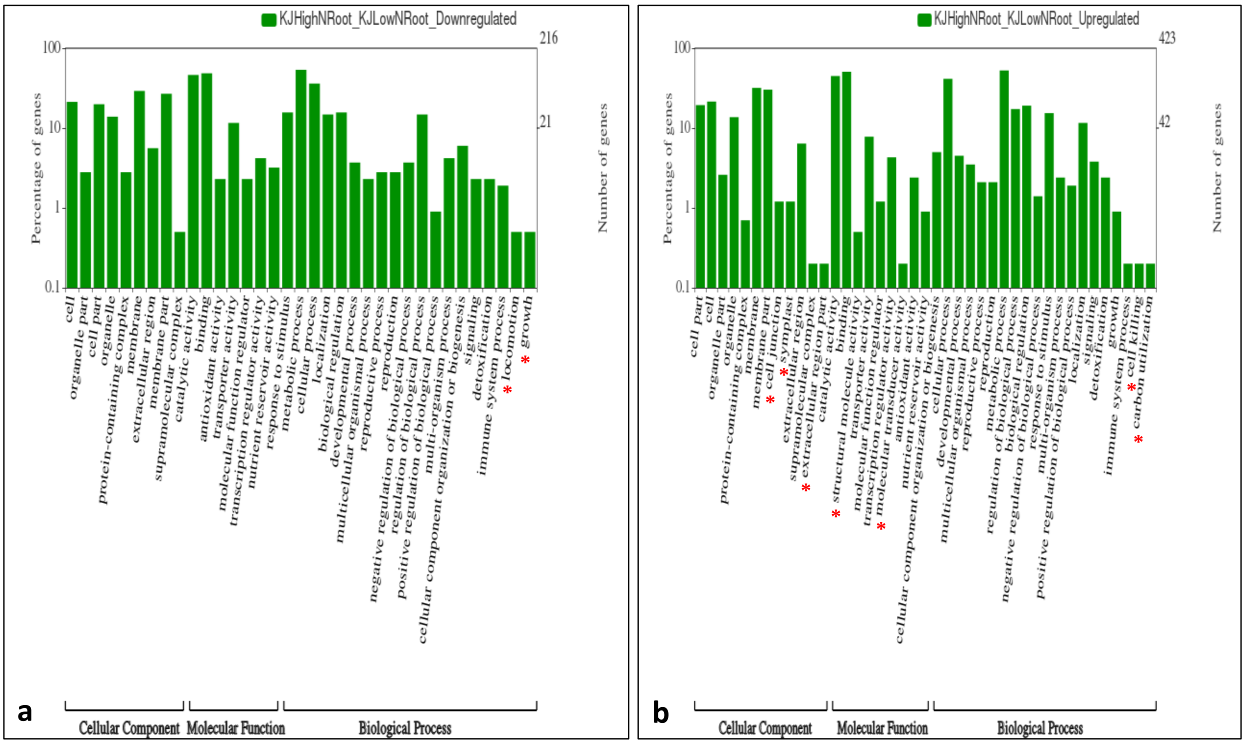

Figure S5. WEGO plot showing the gene ontology (GO) of differentially expressed genes in potato roots grown in aeroponics with low N and high N (control) supply. a) down-regulated genes, and b) up-regulated genes. Red asterisk mark indicates exclusive GO term observed in either down-regulated or up-regulated DEGs.

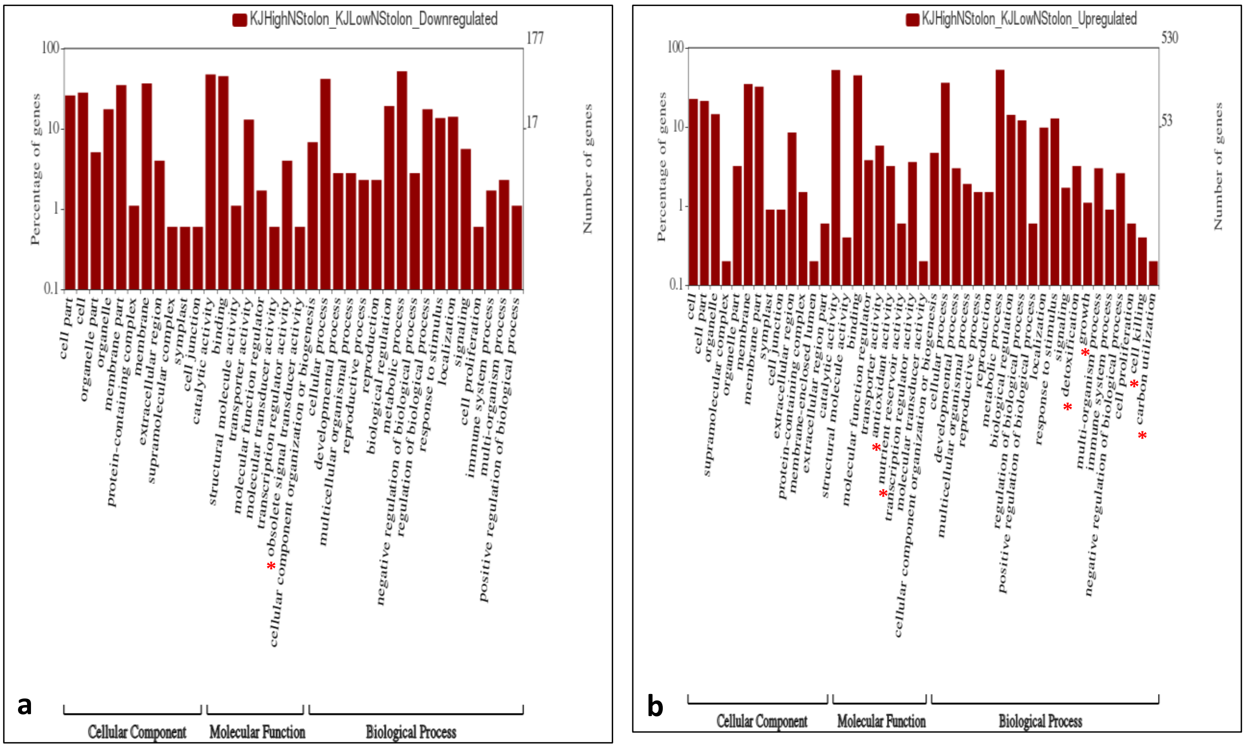

Figure S6. WEGO plot showing the gene ontology (GO) of differentially expressed genes in potato stolons grown in aeroponics with low N and high N (control) supply. a) down-regulated genes, and b) up-regulated genes. Red asterisk mark indicates exclusive GO term observed in either down-regulated or up-regulated DEGs.

Figure S7. Identification of conserved motifs using MEME (version 5.1.0) in highly up-regulated (≥ 3 $\log_2$ fold change) and down-regulated (≤ -3 $\log_2$ fold change) genes with known function in shoots of potato plants grown in aeroponic culture with low N and high N.

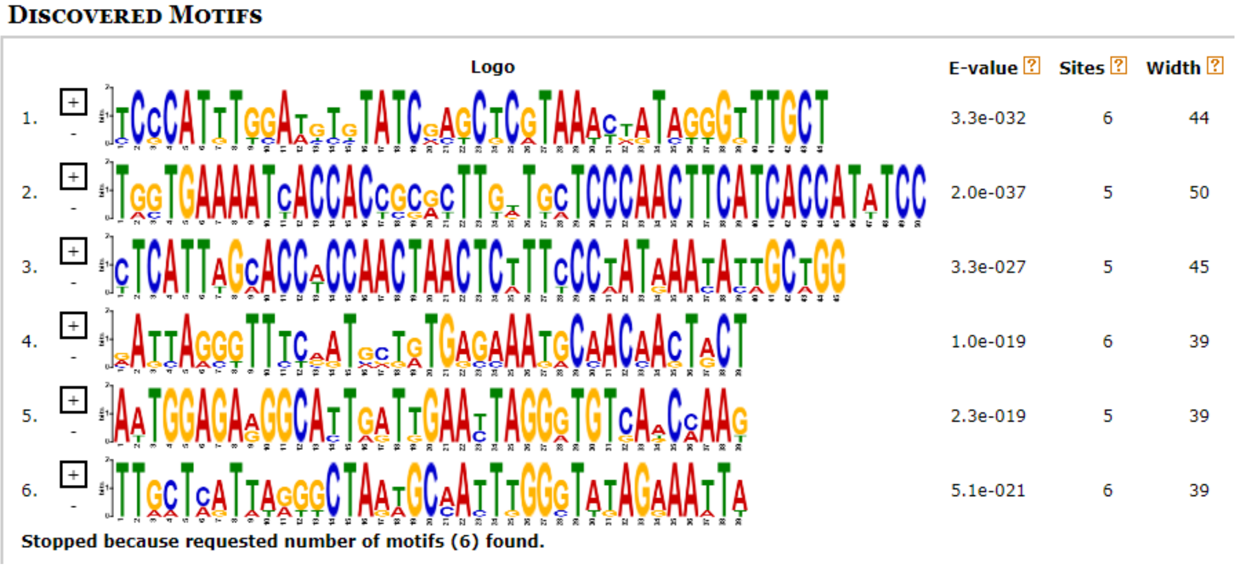

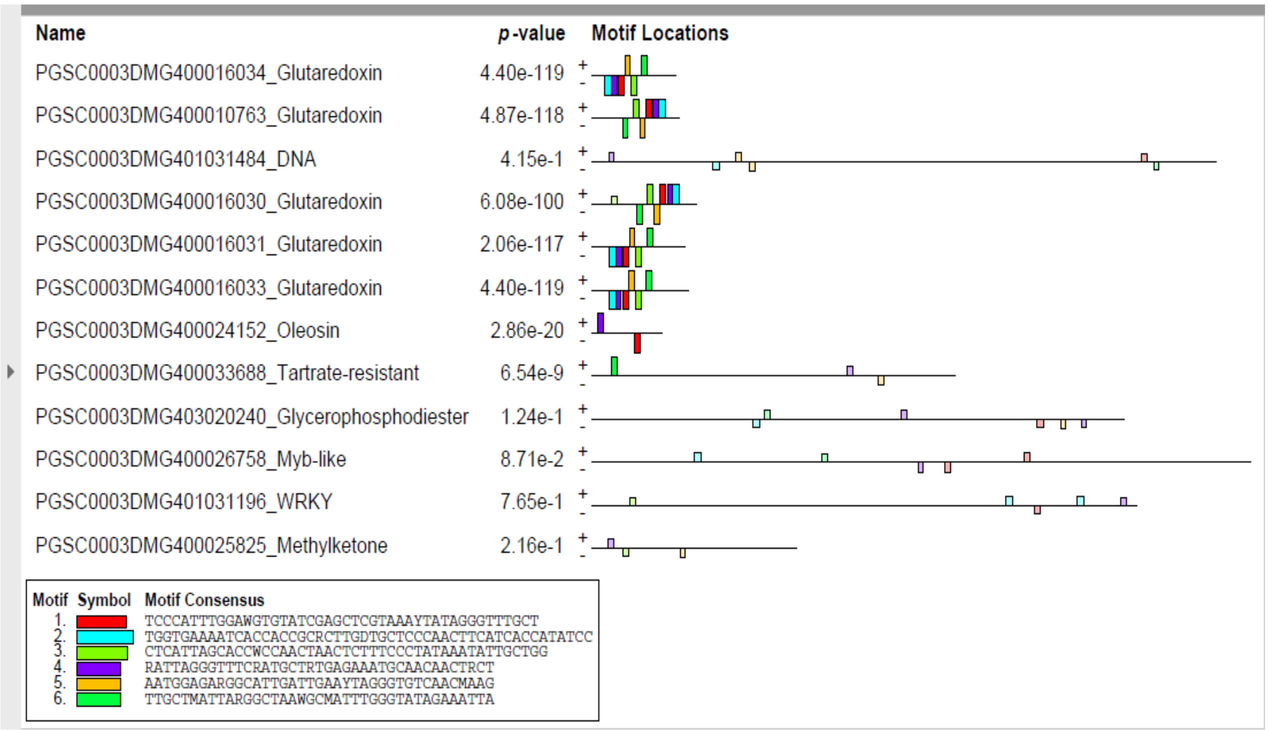

Figure S8. Identification of conserved motifs using MEME (version 5.1.0) in highly up-regulated (≥ 3 $\log_2$ fold change) and down-regulated (≤ -3 $\log_2$ fold change) genes with known function in roots of potato plants grown in aeroponic culture with low N and high N.

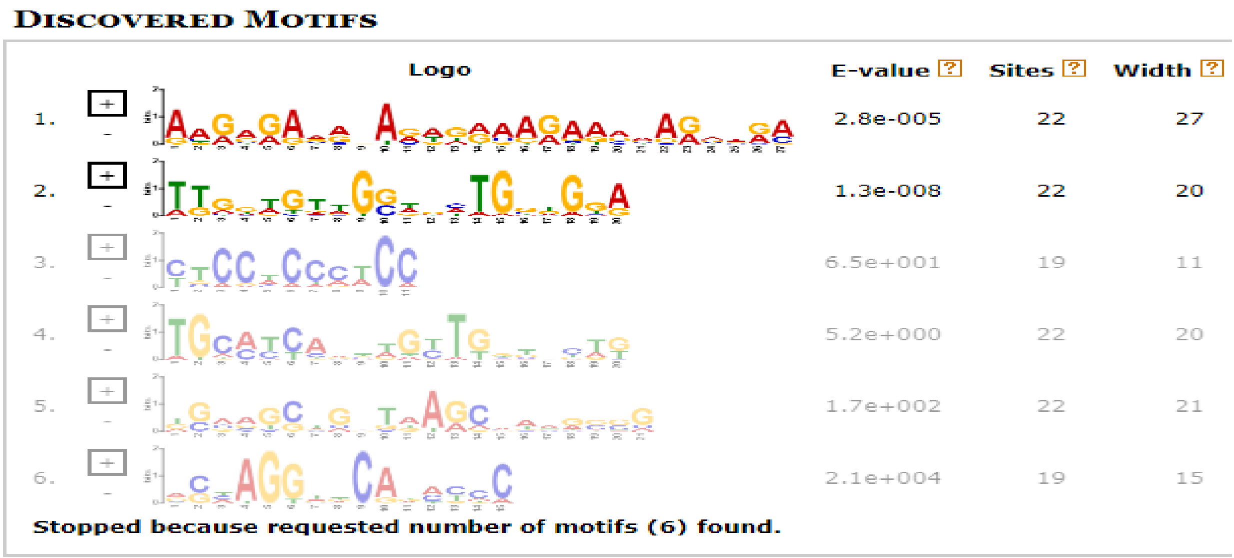

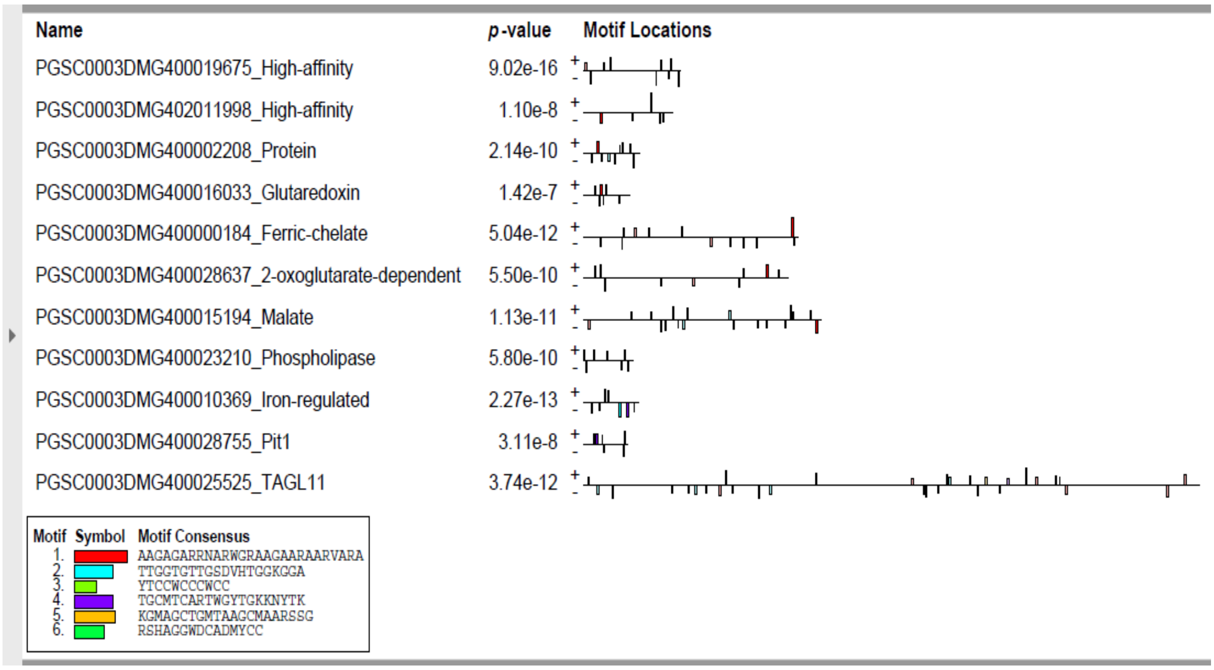

Figure S9. Identification of conserved motifs using MEME (version 5.1.0) in highly up-regulated (≥ 3 $\log_2$ fold change) and down-regulated (≤ -3 $\log_2$ fold change) genes with known function in stolons of potato plants grown in aeroponic culture with low N and high N.

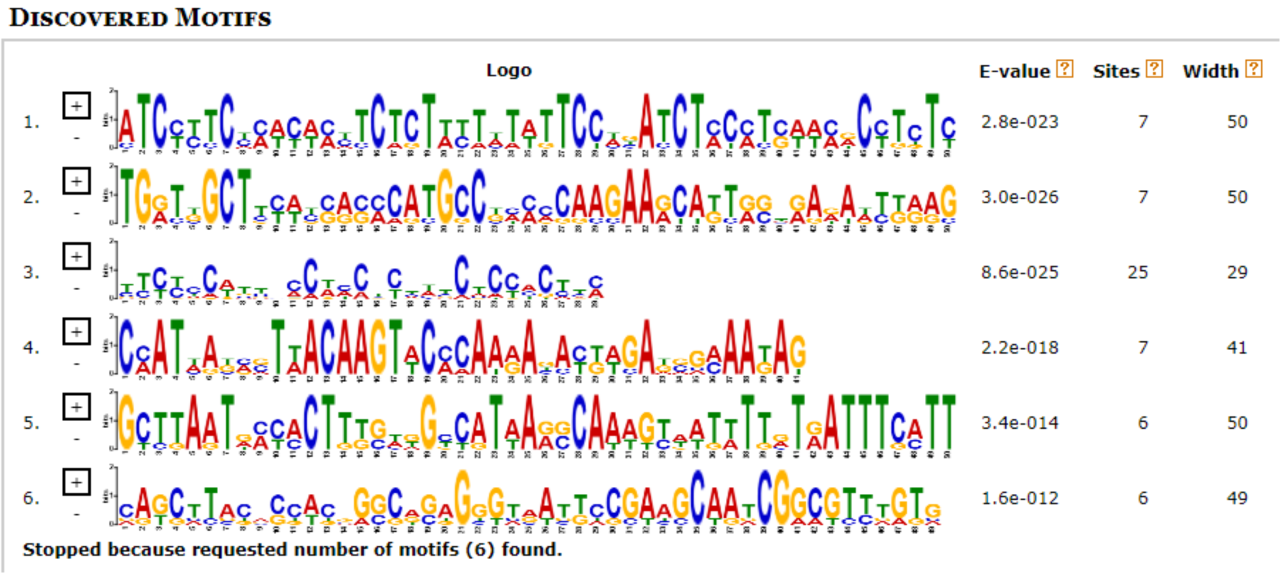

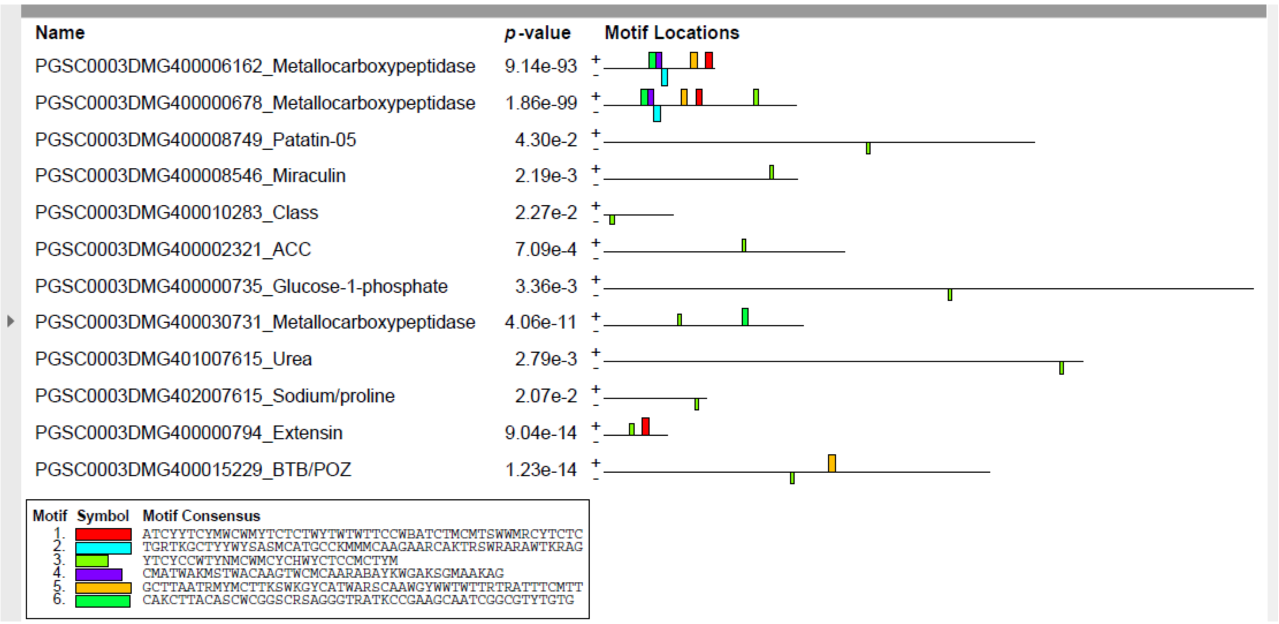

Figure S10. KEGG pathways of N metabolism showing various genes like nitrate transporters, nitrate reductase, nitrite reductase and amino acid synthesis

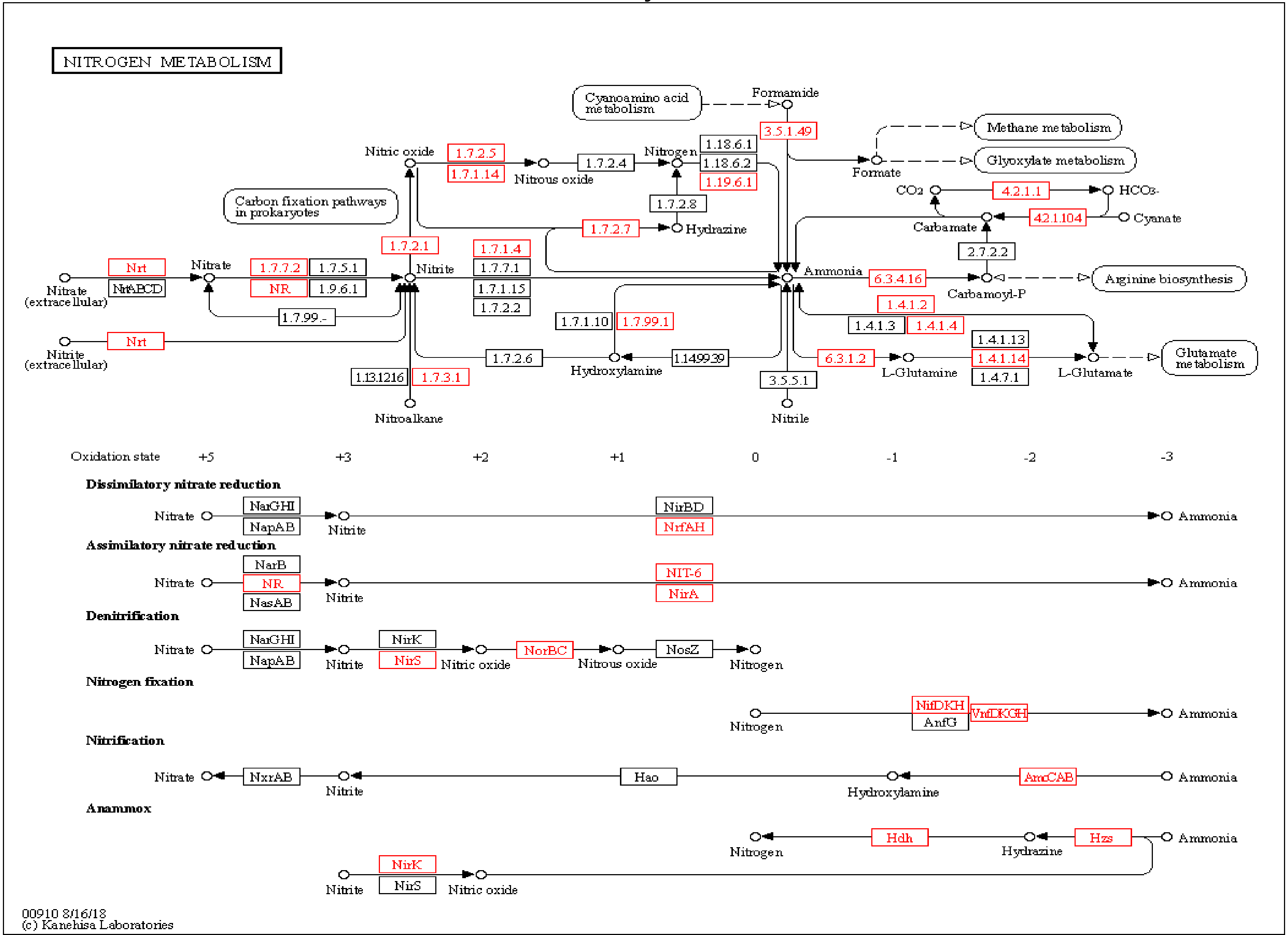

Supplement: Supplementary file 1 — Suppl. Information file. [file 41598_2020_58167_MOESM1_ESM.pdf]
